# The effect of conjugated linoleic acid supplementation in comparison with omega-6 and omega-9 on lipid profile: a graded, dose–response systematic review and meta-analysis of randomized controlled trials

**DOI:** 10.3389/fnut.2024.1336889

**Published:** 2024-03-19

**Authors:** Camellia Akhgarjand, Aryan Tavakoli, Simin Samavat, Amir Bagheri, Aliarash Anoushirvani, Atieh Mirzababaei, Mohammad Reza Amini, Mahmoud Dehghani Ghorbi, Neda Valisoltani, Asieh Mansour, Sayed Mahmoud Sajjadi-Jazi, Hastimansooreh Ansar, Hamid Rezvani

**Affiliations:** ^1^Department of Clinical Nutrition, School of Nutritional Sciences and Dietetics, Tehran University of Medical Sciences, Tehran, Iran; ^2^Department of Cellular and Molecular Nutrition, School of Nutritional Sciences and Dietetics, Tehran University of Medical Sciences, Tehran, Iran; ^3^Department of Community Nutrition, School of Nutritional Sciences and Dietetics, Tehran University of Medical Sciences, Tehran, Iran; ^4^Hemato-Oncology Ward, Firoozgar Hospital, Iran University of Medical Science, Tehran, Iran; ^5^Student Research Committee, Department of Clinical Nutrition and Dietetics, Faculty of Nutrition Sciences and Food Technology, National Nutrition & Food Technology Research Institute, Shahid Beheshti University of Medical Sciences, Tehran, Iran; ^6^Hemato-Oncology Ward, Imam Hossein Hospital, Shahid Beheshti University of Medical Science, Tehran, Iran; ^7^Endocrinology and Metabolism Research Center, Endocrinology and Metabolism Clinical Sciences Institute, Tehran University of Medical Sciences, Tehran, Iran; ^8^Hemato-Oncology Ward, Taleghani Hospital, Shahid Beheshti University of Medical Science, Tehran, Iran

**Keywords:** high-density lipoprotein, conjugated linoleic acids, low-density lipoprotein, meta-analysis, triglycerides

## Abstract

Conjugated linoleic acid (CLA) is a geometrical isomer of linoleic acid, which has anti-inflammatory, anti-diabetic, anti-cancer, and anti-obesity properties. However, the studies reported inconstant results about the CLA-related effects on lipid profiles. As a result, meta-analysis and systematic review were performed to survey the CLA supplementation-related effect on lipid profile including high-density lipoprotein (HDL), low-density lipoprotein (LDL), total cholesterol (TC), and triglycerides (TG). To identify the relevant research, a systematic comprehensive search was initiated on the medical databases such as Scopus and PubMed/Medline until December 2022. The overall effect size was estimated by weighted mean difference (WMD) and 95% confidence interval (CI) in a random effect meta-analysis. In the final quantitative analysis, the meta-analysis considered 35 randomized controlled trials (RCTs) with 1,476 participants (707 controls and 769 cases). The pooled results demonstrated that CLA supplementation, compared with olive oil, significantly increased serum TG levels (WMD: 0.05 mmol/L; 95% CI: 0.01 to 0.1; *p* = 0.04; I^2^ = 0.0%, *p* = 0.91). With regard to TC level, CLA supplementation compared with placebo significantly reduced TC concentrations (WMD: −0.08 mmol/L; 95% CI: −0.14 to −0.02; *p* < 0.001; I^2^ = 82.4%). Moreover, the non-linear dose–response analysis indicated a decreasing trend of TC serum level from the 15th week of CLA supplementation compared with olive oil (P_non-linearity_ = 0.01). The present meta-analysis and systematic review of 35 RCTs showed that the CLA intervention was able to raise the level of TG in comparison to olive oil; however, it can decrease TC level compared with placebo and olive oil.

## Introduction

Dyslipidemia is defined as lipid imbalance such as increased VLDL-C (very low-density lipoprotein-cholesterol), TG (triglycerides), and LDL-C (low-density lipoprotein-cholesterol) concentrations in addition to the reduction in HDL-C (high-density lipoprotein-cholesterol) concentration (1). According to a survey conducted by the United State National Health and Nutrition Examination, 53% of American adults had dyslipidemia (2). Moreover, based on the World Health Organization (WHO), dyslipidemia causes 4 million deaths during a year (1). Epidemiological studies have shown dyslipidemia as a remarkable risk factor in causing hypertension, endothelial dysfunction, primarily cardiovascular disease (CVD), and insulin resistance (3, 4). CVD has been predicted to kill more than 23 million people worldwide (approximately 30.5%) by 2030. CVD is also the main cause of weakness, illness, and death among the Asian population, accounting for nearly 50% of the death rate each year (5, 6). However, dyslipidemia can be corrected with appropriate lifestyle, medical intervention, or a combination of both (7).

It has facilitated the recognition of the fundamental role nutrition plays in the prevention, prognosis, and dyslipidemia treatment (8). Recently, there is an approving attitude toward the health-promoting properties of conjugated linoleic acids (CLAs) (9). CLA is a term that relates to a gaggle of points, and the geometrical isomers of linoleic acid found within some foods including meat, dairy, and ruminant animal fat (10). Conjugation of two bonds of CLA makes the main isomerase in dairy products which is cis9, trans11-18: 2, and other isomerases such as cis9, trans11, and trans10, cis12 are found in industrial dairy products (11). Humans consume 160 mg of CLA in their daily food intake (12). CLA form used in feeding trials is an alkaline isomerase which is a sort of linoleic acid present in vegetable oils containing 9c, 11 t, and 10 t, 12c at the same amount (13). Several health advantages have been investigated for CLA which are equivalent to anti-inflammatory, anti-obesity, anti-diabetic, and anti-cancer properties (14). C9, t11 accounts for 90% of CLA with anti-carcinogenic effects, whereas t10,c12 relates to lipid metabolism with nearly 10% (15). On the other hand, CLA reported some inverse effects on oxidative stress, insulin sensitivity, overweighting in men, and steatosis (16, 17).

Evidence shows a significant improvement in cardiovascular markers by using CLA in experimental studies (11). Moreover, studies demonstrated beneficial effects of CLAs on blood lipid profile (18). In animal studies, the role of CLA in declining body fat, improving lipid, and reversing the development of atherogenic lesions has been investigated (19–22). Kritchevsky et al. reported that supplementation rabbits with CLA decreased atherosclerosis lesions by 30% (13). Another experimental study showed that CLA feeding could significantly reduce LDL-c but not HDL-c (23). The results about the effects of CLA on lipid profile in humans are sparse, and inconclusive some of them showed a direct relationship and others showed a negative relationship. CLA supplementation caused a 60% reduction in TG and VLDL during 8 weeks in both healthy men and women (24). Moloney et al. illustrated that CLA supplementation in humans had a direct effect on HDL-c levels (16). Contrary to this, no changes in lipid profile were reported by other trials. Carvalho et al. indicated no changes in lipid profile after supplementing 3 g/day for 3 months in women with metabolic syndrome (25). Joseph et al. indicated no improvement in CVD markers in healthy men after 8 weeks of CLA intake (26). In addition to this, no effects on blood lipids were found in the Riserus study on obese men (17).

These inconsistencies may come from variations in duration and dose of CLA supplementation or feeding, metabolic status, gender of population, and sample size (27). Overall, it is necessary to summarize all the in-access evidence, applying a comprehensive meta-analysis. Accordingly, this meta-analysis was performed to summarize the available evidence on the effects of CLA supplementation on the levels of adults’ blood lipids.

## Methods

The findings of the present meta-analysis were indicated by employing the preferred reporting items of the Systematic Review and Meta-Analysis (PRISMA) guidelines (28). The protocol of the study has been registered in the International Prospective Register of Systematic Reviews (PROSPERO) database (www.crd.York.ac.uk/PROSPERO, ID = CRD42023409278).

### Search strategy

From inception to September 2023, a comprehensive and systematic search was conducted to identify the relevant studies across medical databases such as Scopus, PubMed/Medline, Google scholar, and EMBASE. The MESH and non-MESH terms applied include: (CLA OR “Linoleic Acid” OR “CLA fatty acid” OR “CLA” OR “trans-10 cis-12-conjugated linoleic acid” OR “conjugated linoleic acid” OR “cis-9 trans-11-conjugated linoleic acid”) AND (RCT OR “Single-Blind Method” OR OR “Cross-Over Studies” OR “Random Allocation” OR “Double-Blind Method” OR “Intervention Studies” OR “Clinical Trials as Topic”) AND (“Hyperlipidemias” OR “Cholesterol, HDL” OR “Lipoproteins, LDL” OR “Dyslipidemias” “Lipoproteins, HDL” OR “Cholesterol, LDL”). There are no language or release date restrictions. In addition, to prevent overlooking the relevant articles, we manually searched the reference lists regarding all relevant studies, even including review articles, published by major journals.

### Eligibility criteria

The PICO criteria (intervention, population, study design, comparison, and outcome), regarding the current meta-analysis, are shown in Table 1. Two inspectors (CA, MA) investigated the online databases to find potentially relevant trials. A chief reviewer (HI) resolved any disputes regarding study selection. The criteria to include the eligible studies were as follows: (1) trials were performed on adults (age older than 18 years); (2) studies evaluated the effect of oral supplementation by conjugated linoleic acid (CLA), at least, on one of the below outputs: HDL, LDL, TC, and TG; and (3) Randomized controlled trials (RCTs) with parallel or crossover design compared with placebo. We excluded the studies that were case–control, cross-sectional, cohort studies, conference papers, and letters, which were conducted on lactating women, children, and pregnant women, and studies in which changes in outcome measures were not clearly or inappropriately reported.

**Table 1:** The population, intervention, comparison, outcome, and study design (PICO) criteria.

Criteria	Description
Population	Adults (aged ≥18 years)
Intervention	Conjugated Linoleic Acid (CLA) supplement
Comparison	Placebo or no intervention
Outcome	Changes in TG, TC, LDL, and HDL serum levels
Study design	Randomized controlled trials

### Data extraction

Irrelevant studies were excluded by two independent authors through reviewing their abstracts and titles. All intervention details included the average age and gender of subjects, study design such as crossover or parallel, author’s first name, subjects’ health status, publication’s year, supplementation duration, and intervention details such as dosage and type of CLA supplement and location of study, and several individuals were included in the placebo and intervention groups. A spreadsheet was standardized to include the mean ± standard deviation and/or changes in the results such as TG, total cholesterol, LDL, and HDL levels before and after supplementation in both cases and controls. The result units, if reported differently, were all converted to the most employed units. A chief investigator (HA) resolved any disagreements.

### Risk of bias

Two independent investigators (CA, MA) examined the bias risk by employing the Cochrane quality assessment tool for RCTs (29). This tool contains seven pre-specified criteria, namely, (a) selective reporting, (b) blinding of personnel and participants, (c) completeness of study outcome information, (d) allocation concealment, (e) random sequence generation, (f) other possible sources of biases, and (g) blinding of study outcome examination. According to this tool, we rated trials in three categories: high quality (low bias risk regarding all domains), fair (high bias risk regarding 1 item), and poor (high bias risk regarding >2 items).

### Statistical analysis

We used Stata software (Stata Crop, College Station Texas, United States) version 14 for all statistical analyses. We used the SDs and mean differences of serum TC, TG, HDL, and LDL serum levels to calculate the overall effect size. DerSimonian and Laird random-effects models were employed to evaluate the overall effect sizes (30) and were expressed as mean differences and 95% confidence interval (CI). To calculate the within-group mean differences for each group, the final mean differences were subtracted from the baseline mean values. The following equation was employed to calculate the standard deviation (SD) of the mean differences: SD change = square root [(SD baseline) 2 + (SD final) 2 – (2 × 0.5 × SD baseline × SD final)] if they were not reported (31). To calculate SD, the following formula was utilized when the trials reported the standard error of the mean (S.E.M): S. D ¼ S.E.Mn (n is the number of subjects in each group). Eventually, two parameters, namely, 95% CI and weighted mean difference, (WMD) were reported as the overall effect size magnitude in a random-effects model. We applied I-square (I^2^) test with a significance level of *p* < 0.10, to determine heterogeneity among the results of included trials. The analyses of subgroups were performed by CLA dosage, trial duration, participants’ mean age, population’s health status, gender, and BMI of subjects to identify the probable heterogeneity source. To identify the impact of studies on the overall effect size, we implemented sensitivity analyses employing the leave-on-out method (i.e., one study was removed, and the analysis was repeated) (32). In addition, the non-linear effect of CLA supplement duration on outcome values was determined through fractional polynomial modeling (33). Egger’s test was used to detect the literature bias, which was conducted in 10 or more studies included in each outcome.

### Grading the evidence

Regarding each meta-analysis, the GRADE approach was applied to score the evidence certainty (34).

## Results

### Study selection

**Figure 1:**
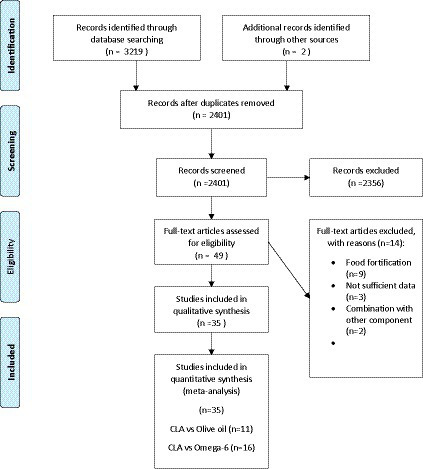
Flow diagram of study selection.

A total of 3,221 citations were identified based on the primary databases (2,340 from Scopus, 879 from PubMed, and 2 from other sources). In total, 820 duplicates were eliminated, and 2,401 articles were left for more examination. Figure 1 indicates the common features of these articles in Summary. The examination of titles and abstracts resulted in excluding of 2,356 studies. The full text of 49 trials was evaluated, which led to exclude other 14 articles due to the following reasons: CLA was used for food fortification purposes (*n* = 9), no sufficient data were reported (*n* = 3), and the effects of CLA were evaluated in the presence of other ingredients (*n* = 2).

Finally, we included 35 RCTs in the final quantitative analysis. Different placebos were used in these studies to better interpret the results; we divided the studies into three groups based on placebo, namely, CLA versus olive oil (*n* = 11) (17, 35–44), CLA versus omega-6 (*n* = 16) (16, 24, 26, 45–57), and CLA versus placebo (*n* = 8) (45, 58–64) and analyzed each separately.

### Study characteristics

Table 2 presents the summary of general features regarding 35 eligible trials. In total, 1,476 participants, including 769 cases and 707 controls, took part in the trials. The publication of articles ranged from 2000 to 2021, which was performed in the United States (46, 49, 55, 56), Sweden Netherlands land (38), Norway (35–37), Korea (39, 40), Brazil (41), United Kingdom (44, 57), IRAN (45, 54, 58, 61–63), Canada (26), South Africa (50), Columbia (53), China (47, 64), Ireland (16, 24), Japan (48), Germany (52), Turkey (59, 60), and Greece (51). The participants’ mean age was between 21.5 and 63.8 years old. The duration of intervention ranged from 4 to 96 weeks, and the CLA supplement dosage was between 1 and 6 g/d. Six studies included only women (39, 41, 46, 52, 54, 60), 8 articles exclusively included men (7, 17, 26, 42, 43, 49, 58, 59), and the rest of them were performed on both genders.

**Table 2:** General characteristics of randomized, double-blind, placebo-controlled parallel trial.

The first author (year)	Country	Health status of subjects	Gender	Participants: CLA/Placebo	Duration (week)	Mean age (year)	Intervention treatment group Placebo		Dosage (g/d)	Outcomes	Results	%variation
*Linoleic acid vs olive oil*												
Blankson et al. (2000)	Norway	Overweight or obese volunteers	F/M	10/8	12	44.3	*cis*-9, *trans*-11 isomer and the *trans*-10, *cis*-12 CLA	Olive oil	6.8	TG,TC, LDL, HDL	Significant reduction of HDL and non-significant change of TG, LDL and TC	NR
Berven et al. (2000)	Norway	Overweight or obese volunteers	F/M	25/22	12	47.6	*cis*-9, *trans*-11 isomer and the *trans*-10, *cis*-12 CLA	Olive oil	3	TG,TC, LDL HDL	Non-significant change of TG, LDL,HDL and TC	NR
Riserus et al. (2001)	Sweden	Men with metabolic syndrome	M	14/10	4	54	Cis9, trans11 CLA 18:2 and trans10, cis12 CLA	Olive oil	4.2	TG, TC, LDL HDL	Non-significant change of TG, LDL,HDL and TC	TG (7.91%), TC (1.27%), LDL (4.52%), HDL (5.3%)
Riserus et al. (2002)	Sweden	Obese Men with metabolic syndrome	M	19/19	12	51	*t*10*c*12 CLA	Olive oil	3.4	TG,TC, LDL, HDL	Non-significant change of TG, LDL,HDL and TC	NR
Kamphuis et al. (2003)	Netherland	Overweight subjects	F/M	14/13	13	40.9	*t*10*c*12 CLA	Olive oil	1. 8	TG	Significant reduction of TG	62.19%
Kamphuis et al. (2003)	Netherland	Overweight subjects	F/M	13/14	13	36.2	*t*10*c*12 CLA	Olive oil	3.6	TG	Significant reduction of TG	61.33%
Riserus et al. (2004)	Sweden	Obese men	M	13/12	12	54	*c*9, *t*11 CLA	Olive oil	3	TG, TC, LDL HDL	Non-significant change of TG, LDL,HDL and TC	NR
Gaullier et al. (2005)	Norway	Overweight subjects	F/M	44/19	96	45.1	CLA–free fatty acid (FFA)	Olive oil	3.6	TG,TC, LDL HDL	Significant reduction of HDL and non-significant change of TG, LDL and TC	TG (−5.6%), TC (−4.26%), LDL (−3.97%), HDL (−4.16%)
Gaullier et al. (2005)	Norway	Overweight subjects	F/M	44/18	96	48.6	CLA-triacylglycerol	Olive oil	3. 4	TG, TC, LDL HDL	Significant reduction of HDL and non-significant change of TG, LDL and TC	TG (−1.53%), TC (−2.97%), LDL (−1.96%), HDL (−5.96)
Taylor et al. (2006)	UK	Healthy volunteers	F/M	21/19	12	45	9c, 11 t CLA, t10, c12, 9c, 11c and 10c, 12c CLA, 9 t, 11 t and 10 t, 11 t and t8, c10 and c11, t13 CLA	Olive oil	4 0.5	TG, TC, LDL HDL	Non-significant change of TG, LDL,HDL and TC	NR
Kim et al. (2008)	Korea	Overweight Korean women	F	15/12	12	26.3	*c*9, *t*11 and *t*10, *c*12 CLA	Olive oil	3	TG, TC, LDL HDL	Non-significant change of TG, LDL,HDL and TC	NR
Park et al. (2008)	South Korea	Healthy overweight/obese	F/M	15/15	8	38.7	t10, c12 CLA	Olive oil	2.4	TG, TC, LDL HDL	Non-significant change of TG, LDL,HDL and TC	NR
Ribeiro et al. (2016)	Brazil	Obese woman	F	15/13	8	23.1	CLA	Olive oil	3.2	TG, TC, LDL HDL	Non-significant change of TG, LDL,HDL and TC	TG (10.47%), TC (1.31%), LDL (1.13%), HDL (−1.39%)
*Linoleic acid vs omega 6*												
Mougios et al. (2001)	Greece	Healthy adults	F/M	10/12	4	22.4	*Soybean*	Soybean oil	1	TG, TC, HDL	Significant reduction of HDL and non-significant change of TG and TC	TG (−13.68%), TC(−3.78%), HDL (−11.97%)
Benito et al. (2001)	USA	Healthy female	F	10/7	9	27	*Cis-, 11 trans-18:2; 8 trans-, 10 cis-18:2; 11 cis-, 13 trans-18:2; and 10 trans-, 12 cis-18:2 CLA*	High-linoleic sunflower oil	3.9	TG, TC, LDL HDL	Non-significant change of TG, LDL,HDL and TC	TG (−40.04%), TC (−6.47%), LDL (−1.06%), HDL (−0.47%)
Noone et al. (2002)	Ireland	Healthy adults	F/M	16/9	8	32.2	*cis-9, trans-11–trans-10, cis-12 (50:50) CLA*	Linoleic acid	3	TG,TC, LDL HDL	Significant reduction of TG and non-significant change of TG, HDL and TC	TG (−20.83%), TC (−1.82%)
Noone et al. (2002)	Ireland	Healthy adults	F/M	17/9	8	28.5	*cis-9, trans-11–trans-10, cis-12 (80:20) CLA*	Linoleic acid	3	TG, TC, LDL HDL	Significant reduction of TG and non-significant change of TG, HDL and TC	TG (−7.40%), TC (−1.79%)
Petridou et al. (2003)	Germany	Healthy nonobese	F	16/16	7	19–24	*Soybean*	Soybean oil	2.1	TG,TC, HDL	Non-significant change of TG, HDL and TC	TG (−9.37%),TC (−5.07%), HDL (−6.16%)
Moloney et al. (2004)	Ireland	Type 2 diabetes mellitus	F/M	16/16	8	63.8	Cis-9, trans-11 and trans-10, cis-12 CLA	Palm oil and soya bean oil	3	TG, TC, LDL HDL	Significant reduction of TC, increase HDL, and non-significant change of TG and HDL	TG (1.23%), TC (−1.93%), LDL (−8.81%), HDL (7.63%)
Whigham et al. (2004)	USA	Healthy obese humans	F/M	27/23	52	43	Cis-9, trans-11 and trans-10, cis-12 CLA	High-linoleic sunflower oil	6	TG, TC, LDL HDL	Significant reduction of TG and non-significant change of TG, HDL and TC	TG (−6.28%), TC (−1/35%), LDL (−4.4%), HDL (0.77%)
Song et al. (2005)	UK	Healthy human	F/M	14/14	12	31.8	CLA	High-linoleic sunflower oil	3	TG, TC, LDL HDL	Significant reduction of TG and TC non-significant change of LDL and HDL	TC (−0.61%),
Watras et al. (2006)	USA	Healthy, overweight subjects	F/M	22/18	24	34	Cis-9, trans-11 and trans-10, cis-12 CLA	Safflower oil	4	TG, TC, LDL HDL	Significant reduction of TC and LDL non-significant change of TG and HDL	NR
Iwata et al. (2007)	Japan	Overweight male	M	20/10	12	44.3	Cis-9, trans-11 and trans-10, cis-12 CLA	Safflower oil	3.4	TG, TC, LDL HDL	Significant reduction of LDL and non-significant change of TG, HDL and TC	TG (−8.86%), TC (−0.38%), LDL (−6.35%0, HDL (2.70%)
Iwata et al. (2007)	Japan	Overweight male	M	20/10	12	40.5	Cis-9, trans-11 and trans-10, cis-12 CLA	Safflower oil	6.8	TG, TC, LDL HDL	Significant reduction of LDL and non-significant change of TG, HDL and TC	TG (−8.46%) TC (0), LDL (−5.93%), HDL (−4.34%)
Steck et al. (2007)	Columbia	Healthy, overweight subjects	F/M	16/8	12	36.3	50:50 ratio of cis-9, trans-11 and trans-10, cis-12 CLA	Safflower oil	3.2	TG, TC, LDL HDL	Non-significant change of TG, LDL,HDL and TC	NR
Steck et al. (2007)	Columbia	Healthy, overweight subjects	F/M	16/8	12	34.1	50:50 ratio of cis-9, trans-11 and trans-10, cis-12 CLA	Safflower oil	6.4	TG, TC, LDL, HDL	Significant reduction of HDL and non-significant change of TG, LDL and TC	NR
Lambert et al. (20 07)	South Africa	Regularly exercising subjects	M	16/16	12	32	Cis-9, trans-11 and trans-12, cis-10 CLA	Safflower oil	3.9	TG,TC, LDL HDL	Significant reduction of TG, LDL,HDL and TC	TG (2.08%), TC (−4.65%), LDL (−9.09), HDL (0)
Lambert et al. (2007)	South Africa	Regularly exercising subjects	F	16/16	12	32	Cis-9, trans-11 and trans-12, cis-10 CLA	Safflower oil	3.9	TG, TC, LDL HDL	Significant reduction of TG, LDL,HDL and TC	TG (−7.40%), TC (−10.20%), LDL (−7.69), HDL (−11.76%)
Tavakoli et al. (2010)	IRAN	Menopause women	F	38/38	12	55.1	Cis 9-trans 11 and trans 10-cis12 CLA	High-linoleic sunflower oil	3.2	TG, TC, LDL HDL	Non-significant change of TG, LDL,HDL and TC	NR
Joseph et al. (2011)	Canada	Overweight male	M	9/5	24	44.8	c9, t11, t10, c12 CLA	Safflower oil	2.8	TG, TC, LDL HDL	Non-significant change of TG, LDL,HDL and TC	NR
Joseph e t.al (2011)	Canada	Overweight male	M	9/4	24	44.8	c9, t11 CLA	Safflower oil	2.7	TG, TC, LDL HDL	Non-significant change of TG, LDL,HDL and TC	NR
Jenkins et al. (2014)	USA	Healthy adults	M	18/16	6	21.5	cis-9, trans-11 isomers and trans-10, cis-12 CLA	High-linoleic sunflower oil	1.4	TG, TC	Non-significant change of TG and TC	TG (−3.19%), TC (0.66)
Chang et al. (2020)	China	Adults with elevated body fat percentage	F/M	32/33	12	25.3	cis-9, trans-11-Octadecadienoic acid: trans-10, cis-12-Octadecadienoic acid CLA	Sunflower oil	3.2	TG, TC, LDL HDL	Non-significant change of TG, LDL,HDL and TC	TG (−7.69%), TG (0), LDL (4.76), HDL (0)
*Linoleic acid vs placebo*												
Colakoglu et al. (2006)	Turkey	Healthy female	F	11/7	6	20.4	CLA	Placebo	3.6	TG, TC, LDL HDL	Non-significant change of TG, LDL,HDL and TC	NR
Cola koglu et al. (2006)	Turkiye	Healthy female	F	12/14	6	21.7	CLA+ Exercise	Exercise	3.6	TG, TC, LDL HDL	Non-significant change of TG, LDL,HDL and TC	NR
Aryaeian et al. (2008)	IRAN	Adults with active rheumatoid arthritis	F/M	22/21	12	43.7	CLA+ vitamin E	Vitamin E	2	TG, HDL, LDL	Non-significant change of TG, LDL and HDL and T	TG (0%), LDL (6.54%), HDL (4.91%)
Aryaeian et al. (2008)	IRAN	Adults with active rheumatoid arthritis	F/M	22/21	12	43.7	CLA	Placebo	2	TG, HDL, LDL	Significant increase LDL and non-significant TG and HDL	TG (0%), LDL (6.54%), HDL (4.91%)
Zhao et al. (2009)	China	Obesity-related hypertension subjects	F/M	40/40	8	62.3	c 9, t 11 and t 10, c 12 CLA	Rmipril	4.5	TG, TC, LD L, HDL	Significant increase of HDL, significant reduction of TC, and non-significant change of TG, LDL	TG (−0.36%), TC (−4.02), LDL (−9.31%), HDL (11.11%)
Bulut et al. (2013)	Turkey	Sedentary male	M	9/9	4	19–31	cis-9, trans-11; trans-10, cis-12 CLA	Inulin	3	TG, TC, LD L, HDL	Significant reduction of TG, LDL and non-significant change of TC and HDL	TG (10.9%), TC (−1.24%), LDL(−15.16%), HDL (7.3%)
Eftekhari et al. (2014)	IRAN	Atherosclerotic adults	F/M	29/28	8	52.79	Cis9, trance11 and trance10, cis12 CLA	Placebo	3	TG, TC, LDL, HDL	Significant ruduction of TC and non-significant change of TG, LDL,HDL	NR
Baghi et al. (2016)	IRAN	Healthy athletes male	M	13/10	12	18.46	CLA	Oral paraffin	5.6	TG, TC, LDL, H DL	Significant ruduction of TC and non-significant change of TG, LDL,HDL	TG (9.84%), TC (−0.83%), LDL (−4.34%), HDL (6.86%)
Fouladi et al. (2018)	IRAN	Overweight adults	F/M	56/58	12	35	cis-9, trans-11 and trans-10, cis-12 CLA	Diet	3	TG,LDL	Significant reduction of TG and LDL and significant increase of HDL	TG (−4.04%), LDL (−2.28%), HDL (5.86%)
Mahdavi et al. (2021)	IRAN	colorectal cancer patients	F/M	15/16	6	62.46	CLA	Placebo	3	TG, TC, LDL,HDL	Significant ruduction of TC and non-significant change of TG, LDL,HDL	TG (47.72%), TC (−6.47%), LDL (−19.76%), HDL (10.1%)

### The assessment bias risk

The bias risk of assessment was conducted by two independent authors (CA and MA) for primary outcomes in 35 qualified RCTs. The summary results are shown in Supplementary Table S2. Based on this assessment, the criteria were only met by one trial regarding low bias risk in all domains (37), and five trials were graded as poor overall quality (41, 46, 61–63). Common biases were concealment of allocation and blinding (assessment of output). The rest of the trials had a fair quality, and for most of them, allocation concealment and blinding (outcome assessment) were unclear.

## Effect of CLA supplement on TG serum level

### CLA supplement versus olive oil

**Figure 2:**
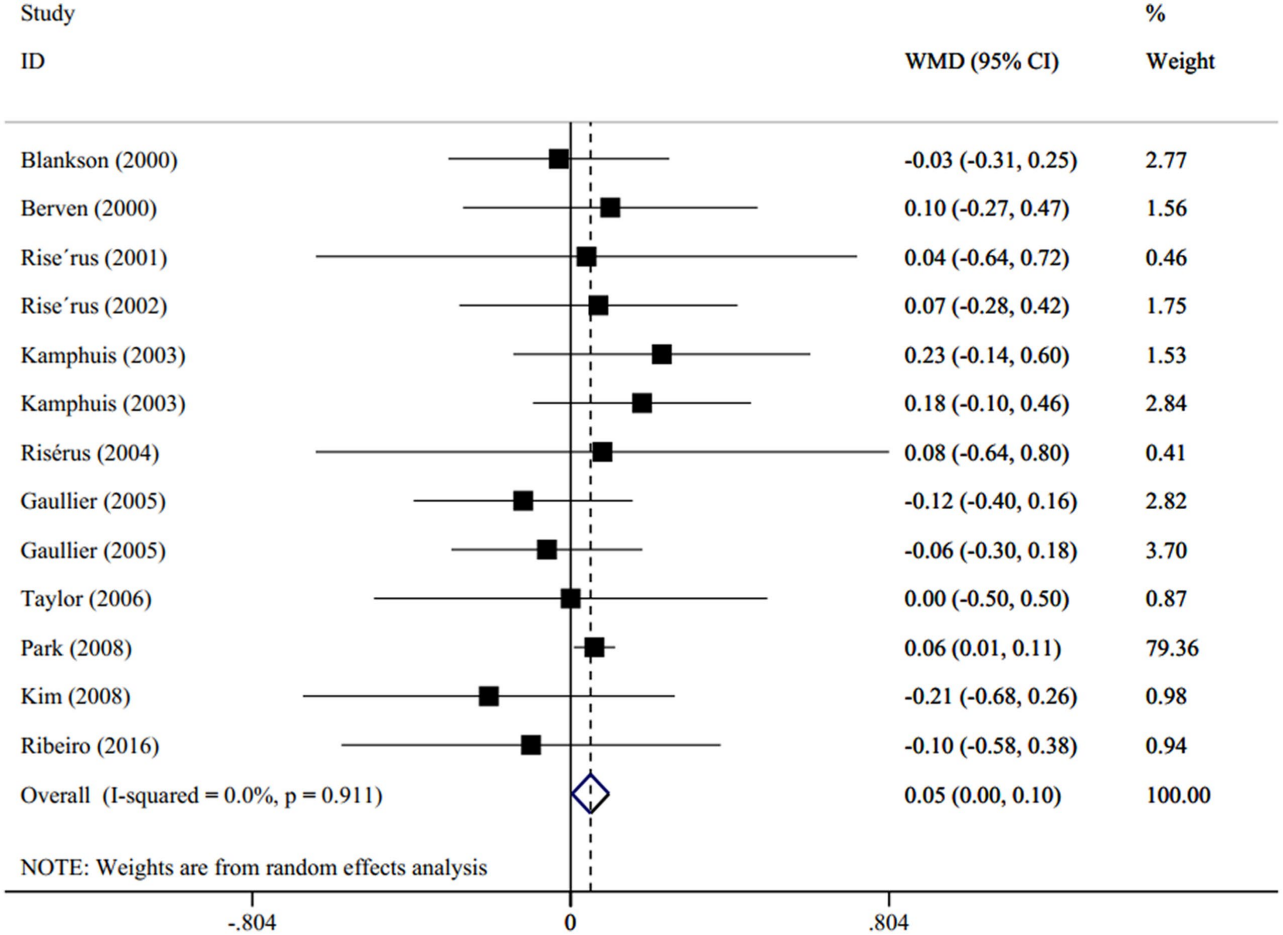
Forest plot for the effect of CLA supplementation versus olive oil on serum levels of TG, expressed as weighted mean differences between the intervention and control groups.

A total of 11 studies (13 effect size; one study used two different types of CLA supplement (37), and one trial used different CLA dosages (38)) including 414 subjects (case = 233 and control = 181) evaluated the effect of the CLA supplement versus olive oil on the levels of serum TG (17, 35–44). The resulting pooled from the random-effects model indicated that the CLA supplementation significantly increased the serum TG levels in proportion to olive oil (WMD: 0.05 mmol/L; 95% CI: 0.00 to 0.1; *p* = 0.04), with no degree of heterogeneity (I^2^ = 0.0%, *p* = 0.91) (Figure 2). Subgroup analysis demonstrated that this increment was observed in the following conditions: (1) dosage < 3.4 g/d (*p* = 0.02), (2) duration ≤12 weeks (*p* = 0.03), (3) age < 45 years old (*p* = 0.018), and BMI < 30 (*p* = 0.03). The detailed subgroup analysis is presented in Supplementary Table S1.

### CLA supplement versus omega-6

Overall, 15 trials (18 effect size; one study reported outcomes in men and women separately (48), and two articles used two different types of CLA supplement (24, 26)) including 608 participants (intervention = 330, control = 278) reported the effect of supplementation with CLA compared with omega-6 on the level of serum TG (16, 24, 26, 45–56). The combination of effect sizes, obtained from the random-effects model, did not significantly result in changes regarding the levels of serum TG after CLA supplement versus omega-6 (WMD: −0.03 mmol/L; 95% CI: −0.08 to 0.01; *p* = 0.45), with a low degree of heterogeneity (I^2^ = 31.6%, *p* = 0.09; Supplementary Figure S1). Subgroup analysis indicated that CLA supplement compared with omega-6 could reduce TG serum levels under the following conditions: (1) age ≥ 35 years old (*p* < 0.001), (2) BMI ≥ 25 (*p* < 0.001), and in male subjects (*p* = 0.025).

### CLA supplement versus placebo

The impact of CLA supplements versus placebo on serum levels of TG was indicated in 8 studies (9 effect size) with 410 subjects (45, 58–64). Polling the effect sizes by the random-effects model indicated no significant effect on serum TG after CLA supplementation (WMD: 0.02 mmol/L; 95% CI: −0.09 to 0.12; *p* = 0.73), with a low degree of heterogeneity (I^2^ = 32.6%, *p* = 0.15; Supplementary Figure S2). Furthermore, after subgroup analyses, no changes were observed in the pooled results.

## Effect of CLA supplement on TC serum level

### CLA supplement versus olive oil

In total, 10 trials (11 effect sizes), including 402 participants, evaluated the effect of CLA supplementation on the levels of serum TC (17, 35–37, 39–44). The levels of serum TC were not affected by CLA supplementation, compared to olive oil, regarding pooled results obtained from the random-effects model (WMD: −0.00 mmol/L; 95% CI: −0.06 to 0.05; *p* = 0.84), with no degree of heterogeneity (I^2^ = 0.0%, *p* = 0.97; Supplementary Figure S3). Moreover, the pooled results did not change across subgroup analyses.

### CLA supplement versus omega-6

The pooled results from 15 trials (18 effect sizes) employing the random-effects model revealed that the alteration in serum TC was not significantly affected by CLA supplementation (WMD: −0.02 mmol/L; 95% CI: −0.15 to 0.12; *p* = 0.17) compared with omega-6. The studies showed a high heterogeneity level (I^2^ = 81.1%, *p* < 0.001; Supplementary Figure S4). Based on subgroup analyses, the potent heterogeneity sources were as follows: dosage of intervention (I^2^ = 10.5%, *p* = 0.34), trial duration (I^2^ = 17.5%, *p* = 0.29), mean age of participants (I^2^ = 0.0%, *p* = 0.63), subject’s BMI (I^2^ = 0.0%, *p* = 0.47), and gender (I^2^ = 0.0%, *p* = 0.92). The CLA supplementation caused a significant increase in the levels of serum TC in men (WMD: 0.07 mmol/L; 95% CI: 0.001 to 0.12; *p* = 0.0.04) compared with omega-6.

### CLA supplement versus placebo

**Figure 3:**
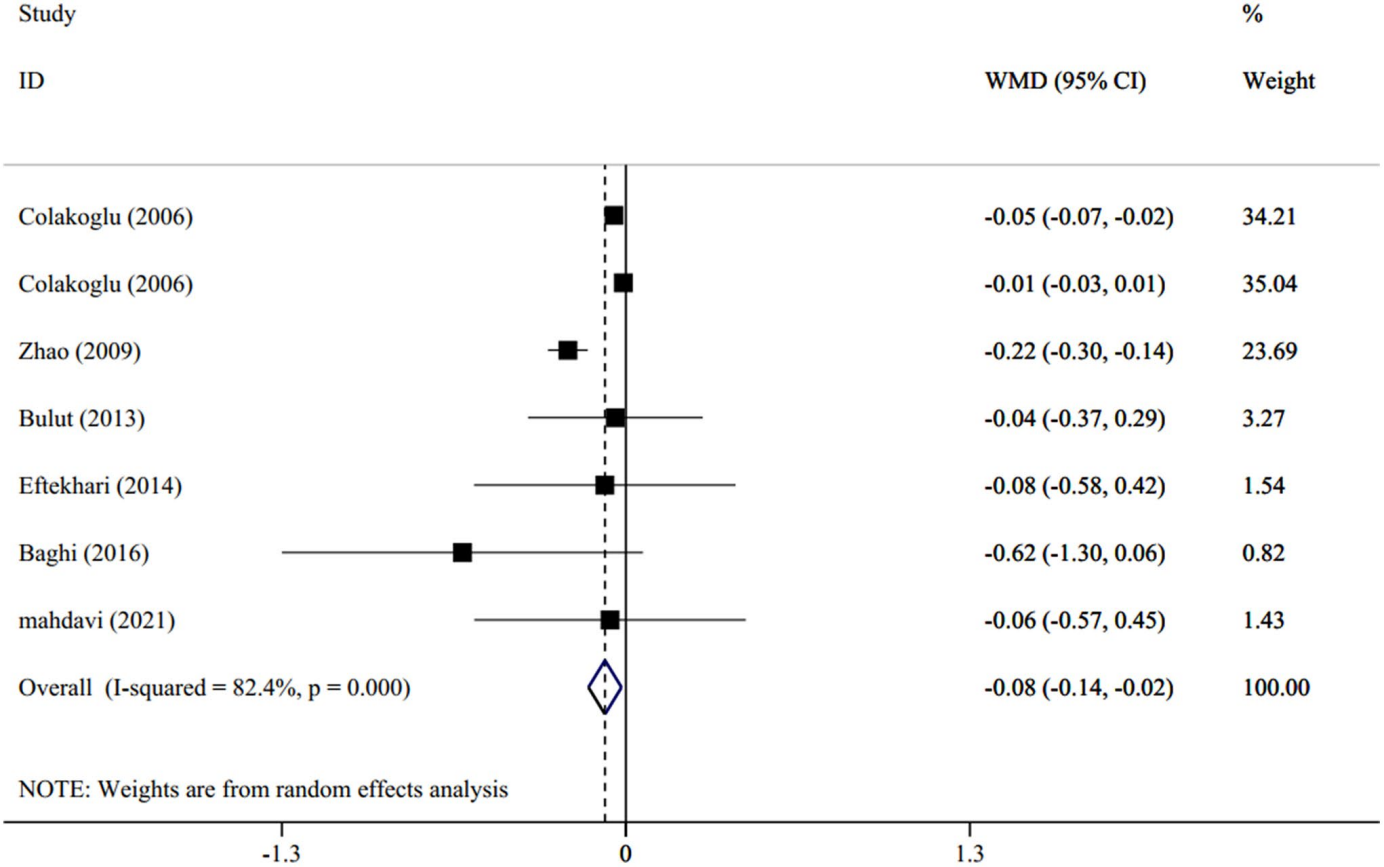
Forest plot for the effect of CLA supplementation versus placebo on serum levels of total cholesterol, expressed as weighted mean differences between the intervention and control groups.

In total, 6 studies (with 7 treatment arms), involving 253 participants, evaluated the effect of CLA supplementation versus placebo on the levels of serum TC. The overall estimates indicated that TC significantly reduced between the intervention and placebo groups (WMD: −0.08 mmol/L; 95% CI: −0.14 to-0.02; *p* < 0.001) with a high heterogeneity between studies (I^2^ = 82.4%, *p* < 0.001) (Figure 3). By subgroup analyses, regarding the dosage of intervention (I^2^ = 0.0%, *p* = 0.99), duration of studies (I^2^ = 0.0%, *p* = 0.44), mean age of subjects (I^2^ = 0.0%, *p* = 0.72), and participant’s BMI (I^2^ = 0.0%, *p* = 0.49), the heterogeneity disappeared. The subgroup analyses indicated that the TC concentration declined significantly, as the introduction of >3 g/d CLA supplements (WMD: −0.08 mmol/L; 95% CI: −0.15 to-0.01; *p* = 0.0.01), lasted for ≥8 weeks (WMD: −0.22 mmol/L; 95% CI: −0.29 to-0.14; *p* < 0.001), was performed on individuals with a mean age of >25 years old (WMD: −0.21 mmol/L; 95% CI: −0.28 to −0.14; *p* < 0.001) and those who had BMI ≥ 25 (WMD: −0.20 mmol/L; 95% CI: −0.28 to −0.13; *p* < 0.001).

## Effect of CLA supplement on LDL serum level

### CLA supplement versus olive oil

There were 10 trials (11 effect sizes) involving 402 participants that compared LDL serum levels between CLA supplementation and olive oil. The pooled effect size employing the random-effects model revealed that the serum LDL levels were not significantly affected by CLA supplementation in comparison to olive oil (WMD: 0.05 mmol/L; 95% CI: −0.03 to 0.13; *p* = 0.36), with no heterogeneity between trials (I^2^ = 0.0%, *p* = 0.97; Supplementary Figure S5). In addition, there were no significant changes after subgroup analyses.

### CLA supplement versus omega-6

The effect of CLA supplementation on the levels of serum LDL, compared with omega-6, was investigated in 11 trials (13 effect sizes), and combining effect sizes from the random-effects model indicated no significant changes following the intervention (WMD: −0.06 mmol/L; 95% CI: −0.18 to 0.05; p = 0.36), with a significant heterogeneity degree (I^2^ = 61%, *p* = 0.002; Supplementary Figure S6). Based on subgroup analyses, the dosage of intervention (I^2^ = 0.0%, *p* = 0.98), the mean age of participants (I^2^ = 0.0%, *p* = 0.92), subject’s BMI (I^2^ = 0.0%, *p* = 0.53), and gender (I^2^ = 0.0%, *p* = 0.70) were the potent heterogeneity sources. Moreover, the analyses reported that CLA supplementation was able to reduce the levels of serum LDL in the trials that employed a low dose of CLA (WMD: −0.16 mmol/L; 95% CI: −0.29 to-0.03; *p* = 0.01) and was performed on people of ≥40 years old (WMD: −0.15 mmol/L; 95% CI: −0.27 to 0.02; *p* = 0.36).

### CLA supplement versus placebo

Eight studies (with 9 effect sizes) revealed the effect of CLA supplementation on the levels of serum LDL compared with placebo. Pooling effect sizes regarding the random-effects model reported that the levels of serum LDL remained unchanged after intervention compared with placebo (WMD: −0.07 mmol/L; 95% CI: −0.29 to 0.14; *p* = 0.64), with a high grade of heterogeneity (I^2^ = 89.9%, *p* < 0.001; Supplementary Figure S7). The heterogeneity was diminished following subgroup analyses, according to the participant’s BMI (I^2^ = 0.0%, *p* = 0.50). The subgroup analyses also revealed a significant reduction in the levels of serum LDL after CLA dosage >3 g/d (WMD: −0.27 mmol/L; 95% CI: −0.51 to −0.03; *p* = 0.02).

## Effect of CLA supplement on the level of HDL serum

### CLA supplement versus olive oil

The effect of CLA supplementation on the levels of serum HDL compared with olive oil was reported in 10 trials (with 11 arm treatments). According to the random-effects model, the levels of serum HDL were not significantly changed (WMD: −0.03 mmol/L; 95% CI: −0.07 to 0.01; *p* = 0.40), with a low grade of heterogeneity (I^2^ = 35.6%, *p* = 0.11; Supplementary Figure S8). Based on the subgroup analyses, CLA supplementation can significantly reduce serum levels of HDL with the following conditions: (1) intervention dosage ≥3.4 g/d (WMD: −0.07 mmol/L; 95% CI: −0.11 to −0.03; *p* < 0.001), trial duration ≥12 weeks (WMD: −0.05 mmol/L; 95% CI: −0.08 to −0.01; *p* = 0.005), subject’s mean age ≥ 45 years old (WMD: −0.05 mmol/L; 95% CI: −0.08 to −0.01; *p* = 0.003), and men (WMD: −0.07 mmol/L; 95% CI: −0.11 to 0.02; *p* = 0.005). However, CLA can increase HDL in BMI ≥ 30 (WMD: 0.06 mmol/L; 95% CI: −0.10 to −0.02; *p* = 0.001).

### CLA supplement versus omega-6

Thirteen trials (containing 15 effect sizes) investigated the effect of CLA supplementation on the levels of serum HDL compared with omega-6. According to the random-effects model, the pooling effect sizes revealed that the CLA supplementation did not significantly affect the levels of serum HDL compared with omega6 (WMD: −0.01 mmol/L; 95% CI: −0.03 to 0.00; *p* = 0.09), with a non-grade of heterogeneity (I^2^ = 0.0%, *p* = 0.88; Supplementary Figure S9). Moreover, the levels of HDL serum remained unchanged after subgroup analyses.

### CLA supplement versus placebo

Overall, 7 studies with 8 intervention arms assessed the effect of CLA supplementation on HDL serum levels compared with placebo. The combination of effect sizes, regarding the random-effects model, demonstrated non-significant changes in HDL serum levels after intervention (WMD: −0.03 mmol/L; 95% CI: −0.05 to 0.12; *p* = 0.14), with a moderate grade of heterogeneity (I^2^ = 65.5%, p = 0.005; Supplementary Figure S10). The subgroup analysis showed the disappearance of between-study heterogeneity in trials using intervention dosage ≤3 g/d (I^2^ = 19.3%, *p* = 0.29), performed on participants of mean age of < 30 years old (I^2^ = 42.3%, *p* = 0.14), subject’s BMI< 25 (I^2^ = 49.8%, *p* = 0.11), and men (I^2^ = 0.0%, *p* = 0.80). Furthermore, this analysis reported a significant decline in the levels of serum HDL after intervention in trials which was performed on women (WMD: −0.75 mmol/L; 95% CI: −1.33 to −0.17; *p* = 0.01).

### Dose–response analysis

**Figure 4:**
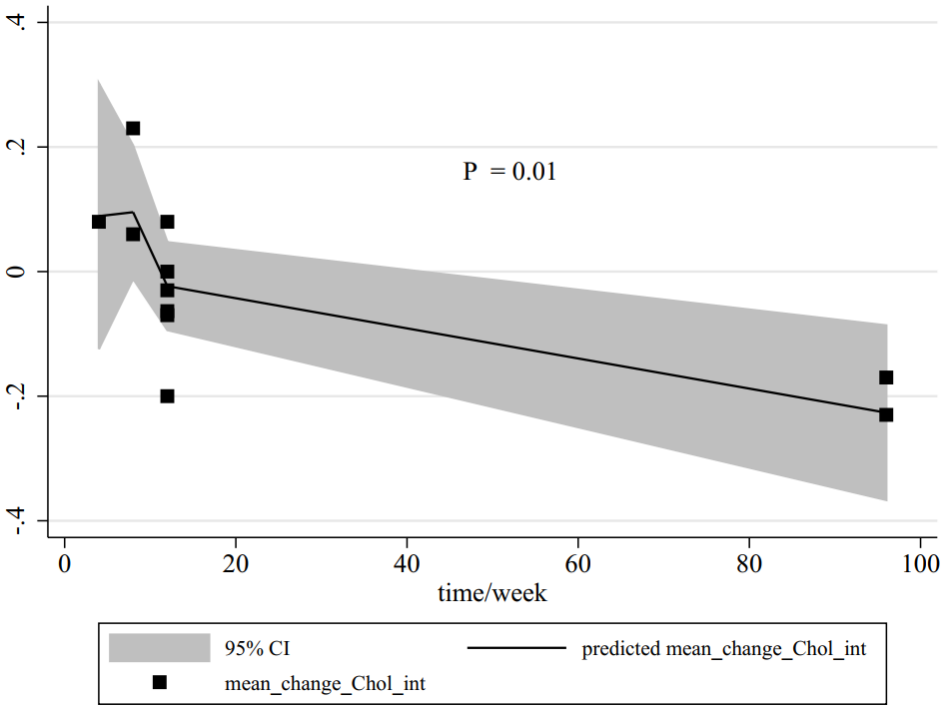
Non-linear dose–response relations between CLA duration (week) and total cholesterol. The 95% CI is revealed in the shaded regions.

Dose–response analysis was performed for all outcomes based on dose and duration, but only this analysis was significant for total cholesterol. Based on the dose–response analysis, a significant non-linear connection was represented between the duration of CLA supplementation compared with olive oil in total cholesterol serum reduction (P_non-linearity_ = 0.01); the reduction trend of total cholesterol serum started from the 15th week (Figure 4).

### Sensitivity analysis

Each study was removed step-wise from the overall analysis, to determine the effect of individual studies on the combined effect size. No single study had a significant effect on the combined effect size of TG and the serum levels of TC, LDL, and HDL.

### Publication bias

Egger’s regression tests refused to verify the publication bias for TG compared with olive oil (*p* = 0.34), TG versus omega-6 (*p* = 0.06), HDL compared with olive oil (*p* = 0.56), and TC in comparison to omega-6 (*p* = 0.55). However, publication bias was confirmed for TC compared with olive oil (p = 0.01) and TC versus omega-6 (*p* = 0.02), LDL compared with olive oil (p = 0.01), and LDL versus omega-6 (*p* = 0.002).

### Grading the evidence

We applied the GRADE approach to rate the evidence certainty. According to the GRADE approach, the evidence certainty was scored very low to low for all outputs, as presented in Supplementary Table S3.

## Discussion

The results of a pooled meta-analysis of 35 RCTs indicated that CLA consumption can increase the level of TG in comparison to olive oil; however, it can decrease TC level in comparison to placebo. This meta-analysis illustrated that CLA supplementation did not change HDL and LDL. As part of this review, a dose–response analysis indicated a significant non-linear connection between the duration of CLA supplementation compared with olive oil in total cholesterol serum reduction; the reduction trend of total cholesterol serum started from the 15th week. Our subgroup analysis revealed that CLA consumption in a dosage of less than 3.4 mg/d with duration of ≤12 weeks in individuals younger than 45 years and BMI <30 kg/m^2^ can significantly increase TG concentration in comparison to olive oil. In animal studies, CLA is reported to have hypotriglyceridemic and anti-atherosclerotic features (65). One trial showed that 8 weeks of CLA supplementation reduced plasma TG and VLDL cholesterol in men and women with normal concentrations of lipids in the blood (24). However, other studies have shown that various doses of CLA supplement form of naturally fortified foods or industrially produced supplements do not affect blood lipids (66, 67). Observed differences between CLA efficacy in animal and human studies may result from differences in supplement dose, duration, species-specific physiology, sex, or initial metabolic state of study samples (27). Mougios et al. (51) reported that 0.7 g/d CLA for 4 weeks caused a reduction in TG levels. Some studies indicated that CLA does not affect serum levels of TG (68). One study reported that CLA supplementation without exercise could lower triacylglycerol concentrations (24), but many studies failed to observe this effect (50, 69, 70). In most studies where CLA supplementation did not affect triglycerides, participants had normal serum triglyceride levels, so probably no changes were observed. On the other hand, olive oil is rich in MUFAs, terpenes, and phenols due to oleic acid, and studies have shown that consuming MUFA-rich dietary fats reduced serum triglyceride levels (71, 72). For this reason, in the current meta-analysis, CLA supplementation appears to increase serum triglyceride levels compared with olive oil.

Our study showed that CLA supplementation may reduce TC concentration when compared with placebo. Moreover, subgroup analysis also revealed that CLA consumption significantly reduced TC concentration in dosage of more than 3 mg/d with duration of ≥8 weeks in subjects older than 25 years and BMI ≥25 kg/m^2^. According to our results, TC levels did not significantly change among non-obese subjects but significantly reduced among obese and overweight subjects. The CLA supplementation in some animal studies lowered cholesterol levels, but in most human studies, total cholesterol and LDL-C were not significantly affected by CLA (11, 70). A recent study in 2022 found that fortifying yogurt with CLA had no significant effect on serum lipids (68). CLA isomers act as PPARγ ligands and enhance their activity. Since peroxisome proliferator-activated receptor gamma (PPAR-γ) is a critical transcription factor in hepatic lipid metabolism, CLA isomers act as PPARγ ligands and enhance its activity and lower serum total cholesterol (73, 74).

Our results represented that CLA supplementation showed no effect on levels of HDL and LDL. Our outcomes are consistent with preceding research that observed no impact of CLA supplementation on HDL and LDL. In most of these studies, subjects were normolipidemic. Subgroup analysis showed that CLA supplementation reduces the level of HDL serum compared with omega-6 and placebo in women. Warensjo et al. (75) indicated that women have significantly higher levels of delta-6 desaturase activities than men, and therefore, they might need higher doses of CLA supplementation than men. This may explain why studies on women showed no significant improvement in HDL-C after CLA supplementation. Furthermore, subgroup analysis showed decreased HDL-C serum concentrations after CLA supplementation in studies using olive oil in the control groups under the following conditions: in men aged ≥45 years, over 12 weeks of supplementation, and dosage ≥3.4 mg/day. A decrease in plasma HDL-C levels has been reported as a side effect of CLA supplementation (76), and Tholstrup and Riserus also reported that CLA supplementation lowered HDL-C levels (43, 77). However, in some research studies, the mixture of CLA isomers failed to affect HDL-C levels (78).

A current meta-analysis reported that CLA supplementation compared with studies that used omega-6 in their control group could only increase TC in men. Most studies have ignored the effects of these vegetable omega-6 oils as a placebo, which may further mislead the results. It has been reported that sunflower oil compared with CLA supplementation reduced serum levels of TC (79). CLA encodes enzymes involved in testosterone biosynthesis such as 17α-hydroxylase/17,20-lyase can upregulate genes, thereby increasing testosterone synthesis (80). Testosterone interference modulates the expression of lipid metabolism (81), which can explain the result.

Another result of the study was that in subjects over 45 years, CLA can reduce serum levels of LDL compared with the group that received omega-6. One study reported that rabbits fed by CLA had significantly lower LDL cholesterol, but no significant changes were observed in the HDL concentrations. In older adults (>45 years), CLA may lower LDL due to altered gene expression. Aging appears to increase the expression of multiple genes that intermediate the inflammatory process, such as the induction of interleukin-6 by NO synthase (iNOS) (82). CLA supplementation by suppressing the iNOS gene expression can reduce IL-6 (83). It should be noted that the mechanism of action of CLA on lipid profile is complex and is not yet fully understood. Moreover, no compromise is advised on CLA-recommended dosage, while the existing evidence proposed 3 g/d as the highly desirable (84).

To the best of our knowledge, it is the first graded, dose–response meta-analysis and systematic review, evaluating the effect of CLA supplementation on lipid profile. Meta-analyses and systematic reviews are at the forefront of clinical evidence (85). However, we observed between-study heterogeneity among the included studies, but subgroup analysis revealed potent heterogeneity sources. Furthermore, the dose–response analysis was another strength of the present review. The current study also had some limitations. Trials were performed on subjects with various health statuses, moreover trials used different doses and different mixtures of CLA isomers that could affect our results. Moreover, publication bias was found in the results for TC versus olive oil, TC versus omega-6, LDL versus olive oil, and LDL versus omega-6.

## Conclusion

The meta-analysis and comprehensive systematic review of placebo-controlled and randomized clinical trials revealed that CLA supplement is not able to modify lipid profile, and it seems necessary to determine the optimal mixture of CLA isomers in people with different health statuses.

## Data availability statement

The original contributions presented in the study are included in the article/Supplementary material, further inquiries can be directed to the corresponding authors.

## Author contributions

CA: Writing – original draft, Writing – review & editing. AT: Writing – review & editing. SS: Methodology, Writing – review & editing. AB: Formal analysis, Writing – original draft. AA: Writing – review & editing. AtM: Writing – original draft. MA: Writing – original draft. MG: Writing – review & editing. NV: Writing – review & editing. AsM: Writing – review & editing. SS-J: Writing – review & editing. HA: Writing – review & editing. HR: Writing – review & editing.

## References

[1] Smith GJ. epidemiology of dyslipidemia and economic burden on the healthcare system. Am J Manag Care. (2007) 13:S68–71. 17596114

[2] Tóth PP Potter D. Prevalence of lipid abnormalities in the United States: the National Health and nutrition examination survey 2003–2006. J Clin Lipidol. (2012) 6:325–30. doi: 10.1016/j.jacl.2012.05.002 22836069

[3] Karam I Ma N Liu X-W Li S-H Kong X-J Li J-Y. Regulation effect of aspirin eugenol Ester on blood lipids in Wistar rats with hyperlipidemia. BMC Vet Res. (2015) 11:217. doi: 10.1186/s12917-015-0523-5 26289078 PMC4546030

[4] Rouhani MH Rashidi-Pourfard N Salehi-Abargouei A Karimi M Haghighatdoost F. Effects of egg consumption on blood lipids: a systematic review and Meta-analysis of randomized clinical trials. J Am Coll Nutr. (2018) 37:99–110. doi: 10.1080/07315724.2017.1366878, PMID: 29111915

[5] Mozaffarian D Benjamin EJ Go AS Arnett DK Blaha MJ Cushman M . Heart disease and stroke statistics—2015 update: a report from the American Heart Association. Circulation. (2015) 131:e29–e322. doi: 10.1161/CIR.0000000000000152, PMID: 25520374

[6] World Health Organization. Global status report on noncommunicable diseases 2010. Hagerstown, MD: Lippincott Williams & Wilkins (2011).

[7] Jawalekar SL Kulkarni UJ Surve VT Deshmukh YA. Status of lipid profile, MDA and protein carbonyl in patients with cardiovascular diseases. Arch Appl Sci Res. (2010) 2:8–14.

[8] Springmann M Wiebe K Mason-D'Croz D Sulser TB Rayner M Scarborough P. Health and nutritional aspects of sustainable diet strategies and their association with environmental impacts: a global modelling analysis with country-level detail. Lancet Planetary Health. (2018) 2:e451–61. doi: 10.1016/S2542-5196(18)30206-7, PMID: 30318102 PMC6182055

[9] Sneddon AA Tsofliou F Fyfe CL Matheson I Jackson DM Horgan G . Effect of a conjugated linoleic acid and ω‐3 fatty acid mixture on body composition and adiponectin. Obes Res. (2008) 16:1019–24. doi: 10.1038/oby.2008.41, PMID: 18356842

[10] Schmid A Collomb M Sieber R Bee GJMS. Conjugated linoleic acid in meat and meat products: a review. Meat Sci. (2006) 73:29–41. doi: 10.1016/j.meatsci.2005.10.010 22062051

[11] Bhattacharya A Banu J Rahman M Causey J Fernandes G. Biological effects of conjugated linoleic acids in health and disease. Nutr Rep Int. (2006) 17:789–810. doi: 10.1016/j.jnutbio.2006.02.009, PMID: 16650752

[12] Chin S Liu W Storkson J Ha Y Pariza MW. Dietary sources of conjugated dienoic isomers of linoleic acid, a newly recognized class of anticarcinogens. J Food Compost Anal. (1992) 5:185–97. doi: 10.1016/0889-1575(92)90037-K

[13] Kritchevsky D Tepper SA Wright S Tso P Czarnecki SK. Influence of conjugated linoleic acid (CLA) on establishment and progression of atherosclerosis in rabbits. J Am Nutr Assoc. (2000) 19:472S–7S. doi: 10.1080/07315724.2000.10718950, PMID: 10963467

[14] Benjamin S Spener FJN. Conjugated linoleic acids as functional food: an insight into their health benefits. Nutr Metab. (2009) 6:36–13. doi: 10.1186/1743-7075-6-36, PMID: 19761624 PMC2754987

[15] Martin J-C Valeille K. Conjugated linoleic acids: all the same or to everyone its own function? Animal. (2002) 42:525–36. doi: 10.1051/rnd:2002042 12625417

[16] Moloney F Yeow T-P Mullen A Nolan JJ Roche HM. Conjugated linoleic acid supplementation, insulin sensitivity, and lipoprotein metabolism in patients with type 2 diabetes mellitus. J Clin Nutr. (2004) 80:887–95. doi: 10.1093/ajcn/80.4.887, PMID: 15447895

[17] Risérus U Vessby B Ärnlöv J Basu S. Effects of cis-9,trans-11 conjugated linoleic acid supplementation on insulin sensitivity, lipid peroxidation, and proinflammatory markers in obese men. J Clin Nutr. (2004) 80:279–83. doi: 10.1093/ajcn/80.2.279, PMID: 15277146

[18] Holub BJJP. Docosahexaenoic acid (DHA) and cardiovascular disease risk factors. Prostaglandins Leukot Essent Fatty Acids. (2009) 81:199–204. doi: 10.1016/j.plefa.2009.05.016 19545988

[19] Park Y Albright KJ Liu W Storkson JM Cook ME Pariza MWJL. Effect of conjugated linoleic acid on body composition in mice. Lipids. (1997) 32:853–8. doi: 10.1007/s11745-997-0109-x 9270977

[20] DeLany JP Blohm F Truett AA Scimeca JA West DB. Conjugated linoleic acid rapidly reduces body fat content in mice without affecting energy intake. Am J Physiol Regul Integr Comp Physiol. (1999) 276:R1172–9. doi: 10.1152/ajpregu.1999.276.4.R1172, PMID: 10198400

[21] Wilson T Nicolosi R Chrysam M Kritchevsky D. Conjugated linoleic acid reduces early aortic atherosclerosis greater than linoleic acid in hypercholesterolemic hamsters. Nutr Res. (2000) 20:1795–805. doi: 10.1016/S0271-5317(00)00268-2

[22] Toomey S Roche H Fitzgerald D Belton OJBST. Regression of pre-established atherosclerosis in the apoE−/− mouse by conjugated linoleic acid. Biochem Soc Trans. (2003) 31:1075–9. doi: 10.1042/bst0311075, PMID: 14505483

[23] Nakamura YK Flintoff-Dye N Omaye ST. Conjugated linoleic acid modulation of risk factors associated with atherosclerosis. Nutr Metab. (2008) 5:1–20. doi: 10.1186/1743-7075-5-22, PMID: 18718021 PMC2546407

[24] Noone EJ Roche HM Nugent AP Gibney MJ. The effect of dietary supplementation using isomeric blends of conjugated linoleic acid on lipid metabolism in healthy human subjects. Brit J Nutr. (2002) 88:243–51. doi: 10.1079/BJN2002615, PMID: 12207834

[25] Carvalho RF Uehara SK Rosa GJVH Management R. Microencapsulated conjugated linoleic acid associated with hypocaloric diet reduces body fat in sedentary women with metabolic syndrome. Vasc Health Risk Manag. (2012) 8:661–7. doi: 10.2147/VHRM.S37385, PMID: 23271912 PMC3526145

[26] Joseph SV Jacques H Plourde M Mitchell PL McLeod RS Jones PJH. Conjugated linoleic acid supplementation for 8 weeks does not affect body composition, lipid profile, or safety biomarkers in overweight, hyperlipidemic men. J Nutr. (2011) 141:1286–91. doi: 10.3945/jn.110.135087, PMID: 21593349

[27] Plourde M Jew S Cunnane SC Jones PJJNR. Conjugated linoleic acids: why the discrepancy between animal and human studies? Nutrit Rev. (2008) 66:415–21. doi: 10.1111/j.1753-4887.2008.00051.x, PMID: 18667017

[28] Shamseer L Moher D Clarke M Ghersi D Liberati A Petticrew M . Preferred reporting items for systematic review and meta-analysis protocols (PRISMA-P) 2015: elaboration and explanation. BMJ. (2015) 350:349. doi: 10.1136/bmj.g7647, PMID: 25555855

[29] Higgins JP Altman DG Gotzsche PC Juni P Moher D Oxman AD . The Cochrane Collaboration’s tool for assessing risk of bias in randomised trials. BMJ. (2011) 343:d5928. doi: 10.1136/bmj.d5928, PMID: 22008217 PMC3196245

[30] DerSimonian R Kacker R. Random-effects model for meta-analysis of clinical trials: an update. Contemp Clin Trials. (2007) 28:105–14. doi: 10.1016/j.cct.2006.04.004 16807131

[31] Borenstein M Hedges L Higgins J Rothstein H. Introduction to meta-analysis John Wiley & Sons (2011).

[32] Sahebkar A. Are Curcuminoids effective C-reactive protein-lowering agents in clinical practice? Evidence from a Meta-analysis. Phytother Res. (2014) 28:633–42. doi: 10.1002/ptr.5045, PMID: 23922235

[33] Fan J Gijbels I. Local polynomial modelling and its applications Routledge (2018).

[34] Guyatt GH Oxman AD Vist GE Kunz R Falck-Ytter Y Alonso-Coello P . GRADE: an emerging consensus on rating quality of evidence and strength of recommendations. BMJ. (2008) 336:924–6. doi: 10.1136/bmj.39489.470347.AD, PMID: 18436948 PMC2335261

[35] Berven G Bye A Hals O Blankson H Fagertun H Thom E . Safety of conjugated linoleic acid (CLA) in overweight or obese human volunteers. Eur J Lipid Sci Technol. (2000) 102:455–62. doi: 10.1002/1438-9312(200008)102:7<455::AID-EJLT455>3.0.CO;2-V

[36] Blankson H Stakkestad JA Fagertun H Thom E Wadstein J Gudmundsen O. Conjugated linoleic acid reduces body fat mass in overweight and obese humans. J Nutr. (2000) 130:2943–8. doi: 10.1093/jn/130.12.2943, PMID: 11110851

[37] Gaullier J-M Halse J Høye K Kristiansen K Fagertun H Vik H . Supplementation with conjugated linoleic acid for 24 months is well tolerated by and reduces body fat mass in healthy, overweight humans. J Nutr. (2005) 135:778–84. doi: 10.1093/jn/135.4.778, PMID: 15795434

[38] Kamphuis MM Lejeune MP Saris WH Westerterp-Plantenga MS. The effect of conjugated linoleic acid supplementation after weight loss on body weight regain, body composition, and resting metabolic rate in overweight subjects. Int J Obes. (2003) 27:840–7. doi: 10.1038/sj.ijo.0802304, PMID: 12821971

[39] Kim J-H Kim O-H Ha Y-L Kim J-O. Supplementation of conjugated linoleic acid with γ-oryzanol for 12 weeks effectively reduces body fat in healthy overweight Korean women. Prev Nutr Food Sci. (2008) 13:146–56. doi: 10.3746/jfn.2008.13.3.146

[40] Park E-J Kim J-M Kim K-T Paik H-D. Conjugated linoleic acid (CLA) supplementation for 8 weeks reduces body weight in healthy overweight/obese Korean subjects. Food Sci Biotechnol. (2008) 17:1261–4.

[41] Ribeiro AS Pina FLC Dodero SR Silva DR Schoenfeld BJ Sugihara P . Effect of conjugated linoleic acid associated with aerobic exercise on body fat and lipid profile in obese women: a randomized, double-blinded, and placebo-controlled trial. Int J Sport Nutr Exerc Metab. (2016) 26:135–44. doi: 10.1123/ijsnem.2015-0236, PMID: 26402730

[42] Risérus U Arner P Brismar K Vessby B. Treatment with Dietarytrans10cis12 conjugated linoleic acid causes isomer-specific insulin resistance in obese men with the metabolic syndrome. Diabetes Care. (2002) 25:1516–21. doi: 10.2337/diacare.25.9.1516, PMID: 12196420

[43] Riserus U Berglund L Vessby B. Conjugated linoleic acid (CLA) reduced abdominal adipose tissue in obese middle-aged men with signs of the metabolic syndrome: a randomised controlled trial. Int J Obes. (2001) 25:1129–35. doi: 10.1038/sj.ijo.0801659, PMID: 11477497

[44] Taylor JS Williams SR Rhys R James P Frenneaux MP. Conjugated linoleic acid impairs endothelial function. Arterioscler Thromb Vasc Biol. (2006) 26:307–12. doi: 10.1161/01.ATV.0000199679.40501.ac, PMID: 16339498

[45] Aryaeian N Shahram F Djalali M Eshragian MR Djazayeri A Sarrafnejad A . Effect of conjugated linoleic acid, vitamin E and their combination on lipid profiles and blood pressure of Iranian adults with active rheumatoid arthritis. Vasc Health Risk Manag. (2008) 4:1423–32. doi: 10.2147/VHRM.S3822, PMID: 19337555 PMC2663461

[46] Benito P Nelson G Kelley D Bartolini G Schmidt P Simon V. The effect of conjugated linoleic acid on plasma lipoproteins and tissue fatty acid composition in humans. Lipids. (2001) 36:229–36. doi: 10.1007/s11745-001-0712-x, PMID: 11337977

[47] Chang H Gan W Liao X Wei J Lu M Chen H . Conjugated linoleic acid supplements preserve muscle in high-body-fat adults: a double-blind, randomized, placebo trial. Nutr Metab Cardiovasc Dis. (2020) 30:1777–84. doi: 10.1016/j.numecd.2020.05.029, PMID: 32684362

[48] Iwata T Kamegai T Yamauchi-Sato Y Ogawa A Kasai M Aoyama T . Safety of dietary conjugated linoleic acid (CLA) in a 12-weeks trial in healthy overweight Japanese male volunteers. J Oleo Sci. (2007) 56:517–25. doi: 10.5650/jos.56.517, PMID: 17898458

[49] Jenkins ND Buckner SL Cochrane KC Bergstrom HC Goldsmith JA Weir JP . CLA supplementation and aerobic exercise lower blood triacylglycerol, but have no effect on peak oxygen uptake or cardiorespiratory fatigue thresholds. Lipids. (2014) 49:871–80. doi: 10.1007/s11745-014-3929-0 25034667

[50] Lambert EV Goedecke JH Bluett K Heggie K Claassen A Rae DE . Conjugated linoleic acid versus high-oleic acid sunflower oil: effects on energy metabolism, glucose tolerance, blood lipids, appetite and body composition in regularly exercising individuals. Br J Nutr. (2007) 97:1001–11. doi: 10.1017/S0007114507172822, PMID: 17381964

[51] Mougios V Matsakas A Petridou A Ring S Sagredos A Melissopoulou A . Effect of supplementation with conjugated linoleic acid on human serum lipids and body fat. J Nutr Biochem. (2001) 12:585–94. doi: 10.1016/S0955-2863(01)00177-2 12031264

[52] Petridou A Mougios V Sagredos A. Supplementation with CLA: isomer incorporation into serum lipids and effect on body fat of women. Lipids. (2003) 38:805–11. doi: 10.1007/s11745-003-1129-2, PMID: 14577658

[53] Steck SE Chalecki AM Miller P Conway J Austin GL Hardin JW . Conjugated linoleic acid supplementation for twelve weeks increases lean body mass in obese humans. J Nutr. (2007) 137:1188–93. doi: 10.1093/jn/137.5.1188, PMID: 17449580

[54] Tavakkoli Darestani A Hosseinpanah F Hedayati M Amiri Z Tavakkoli Darestani R Tahbaz F. Conjugated linoleic acid and lipid profile of postmenopausal women. Res Med. (2010) 34:26–34.

[55] Watras A Buchholz A Close R Zhang Z Schoeller D. The role of conjugated linoleic acid in reducing body fat and preventing holiday weight gain. Int J Obes. (2007) 31:481–7. doi: 10.1038/sj.ijo.0803437, PMID: 16924272

[56] Whigham L O’shea M Mohede I Walaski H Atkinson R. Safety profile of conjugated linoleic acid in a 12-month trial in obese humans. Food Chem Toxicol. (2004) 42:1701–9. doi: 10.1016/j.fct.2004.06.008, PMID: 15354322

[57] Song H Grant I Rotondo D Mohede I Sattar N Heys S . Effect of CLA supplementation on immune function in young healthy volunteers. Eur J Clin Nutr. (2005) 59:508–17. doi: 10.1038/sj.ejcn.1602102, PMID: 15674307

[58] Baghi AN Mazani M Nemati A Amani M Alamolhoda S Mogadam RA. Anti-inflammatory effects of conjugated linoleic acid on young athletic males. J Pak Med Assoc. (2016) 66:280–4. 26968277

[59] Bulut S Bodur E Colak R Turnagol H. Effects of conjugated linoleic acid supplementation and exercise on post-heparin lipoprotein lipase, butyrylcholinesterase, blood lipid profile and glucose metabolism in young men. Chem Biol Interact. (2013) 203:323–9. doi: 10.1016/j.cbi.2012.09.022, PMID: 23073171

[60] Colakoglu S Colakoglu M Taneli F Cetinoz F Turkmen M. Cumulative effects of conjugated linoleic acid and exercise on endurance development. J Sports Med Phys Fitness. (2006) 46:4. 17119522

[61] Eftekhari MH Aliasghari F Beigi MAB Hasanzadeh J. The effect of conjugated linoleic acids and omega-3 fatty acids supplementation on lipid profile in atherosclerosis. Adv Biomed Res. (2014) 3:15. doi: 10.4103/2277-9175.124644 24600599 PMC3929013

[62] Fouladi H Peng LS Mohaghehgi A. Effects of conjugated linoleic acid supplementation and exercise on body fat mass and blood lipid profiles among overweight Iranians. Malays J Nutr. (2018) 24

[63] Mahdavi R Mohammadzadeh M Sanaie-Oskouei S Faramarzi E. The effects of conjugated linoleic acid on serum fatty acids composition and lipid profile in patients with rectal Cancer undergoing Chemoradiotherapy. J Isfahan Med School. (2021) 38:996–1003.

[64] Zhao W-S Zhai J-J Wang Y-H Xie P-S Yin X-J Li L-X . Conjugated linoleic acid supplementation enhances antihypertensive effect of ramipril in Chinese patients with obesity-related hypertension. Am J Hypertens. (2009) 22:680–6. doi: 10.1038/ajh.2009.56, PMID: 19300423

[65] McLeod RS LeBlanc AM Langille MA Mitchell PL Currie DL. Conjugated linoleic acids, atherosclerosis, and hepatic very-low-density lipoprotein metabolism. Am J Clin Nutr. (2004) 79:1169S–74S. doi: 10.1093/ajcn/79.6.1169S, PMID: 15159253

[66] Miller A Stanton C Devery R. Cis 9, trans 11-and trans 10, cis 12-conjugated linoleic acid isomers induce apoptosis in cultured SW480 cells. Anticancer Res. (2002) 22:3879–87. PMID: 12553008

[67] Venkatramanan S Joseph SV Chouinard PY Jacques H Farnworth ER Jones PJ. Milk enriched with conjugated linoleic acid fails to alter blood lipids or body composition in moderately overweight, borderline hyperlipidemic individuals. J Am Coll Nutr. (2010) 29:152–9. doi: 10.1080/07315724.2010.10719829, PMID: 20679151

[68] Reynolds C Roche H. Conjugated linoleic acid and inflammatory cell signalling. Prostaglandins Leukot Med. (2010) 82:199–204. doi: 10.1016/j.plefa.2010.02.021, PMID: 20207526

[69] Risérus U Smedman A Basu S Vessby B. CLA and body weight regulation in humans. Lipids. (2003) 38:133–7. doi: 10.1007/s11745-003-1043-7 12733745

[70] Smedman A Vessby B. Conjugated linoleic acid supplementation in humans—metabolic effects. Lipids. (2001) 36:773–81. doi: 10.1007/s11745-001-0784-7, PMID: 11592727

[71] Namayandeh SM Kaseb F Lesan S. Olive and sesame oil effect on lipid profile in hypercholesterolemic patients, which better? Int J Prev Med. (2013) 4:1059–62. PMID: 24130948 PMC3793488

[72] Kaseb F Biregani AN. Effects of olive oil and grape seed oil on lipid profile and blood pressure in patients with hyperlipidemia: a randomized clinical trial. Food Nutr Sci. (2016) 7:682–8. doi: 10.4236/fns.2016.78069

[73] Lowell BB. An essential regulator of adipogenesis and modulator of fat cell function: PPARγ. Cell. (1999) 99:239–42. doi: 10.1016/S0092-8674(00)81654-2, PMID: 10555139

[74] Moya-Camarena SY Heuvel JPV Blanchard SG Leesnitzer LA Belury MA. Conjugated linoleic acid is a potent naturally occurring ligand and activator of PPARα. J Lipid Res. (1999) 40:1426–33. doi: 10.1016/S0022-2275(20)33384-8 10428978

[75] Warensjö E Öhrvall M Vessby B. Fatty acid composition and estimated desaturase activities are associated with obesity and lifestyle variables in men and women. Nutr Metab Cardiovasc Dis. (2006) 16:128–36. doi: 10.1016/j.numecd.2005.06.001, PMID: 16487913

[76] Benjamin S Prakasan P Sreedharan S Wright ADG Spener F. Pros and cons of CLA consumption: an insight from clinical evidences. Nutr Metab. (2015) 12:4–21. doi: 10.1186/1743-7075-12-4, PMID: 25972911 PMC4429457

[77] Raff M Tholstrup T Basu S Nonboe P Sørensen MT Straarup EM. A diet rich in conjugated linoleic acid and butter increases lipid peroxidation but does not affect atherosclerotic, inflammatory, or diabetic risk markers in healthy young men. J Nutr. (2008) 138:509–14. doi: 10.1093/jn/138.3.509 18287358

[78] Gaullier J-M Berven G Blankson H Gudmundsen O. Clinical trial results support a preference for using CLA preparations enriched with two isomers rather than four isomers in human studies. Lipids. (2002) 37:1019–25. doi: 10.1007/s11745-002-0995-y, PMID: 12558050

[79] Asp ML Collene AL Norris LE Cole RM Stout MB Tang S-Y . Time-dependent effects of safflower oil to improve glycemia, inflammation and blood lipids in obese, post-menopausal women with type 2 diabetes: a randomized, double-masked, crossover study. Clin Nutr. (2011) 30:443–9. doi: 10.1016/j.clnu.2011.01.001, PMID: 21295383 PMC3115398

[80] Barone R Macaluso F Catanese P Marino Gammazza A Rizzuto L Marozzi P . Endurance exercise and conjugated linoleic acid (CLA) supplementation up-regulate CYP17A1 and stimulate testosterone biosynthesis. PLoS One. (2013) 8:e79686. doi: 10.1371/journal.pone.0079686, PMID: 24223995 PMC3818175

[81] Bellido T Jilka R Boyce B Girasole G Broxmeyer H Dalrymple S . Regulation of interleukin-6, osteoclastogenesis, and bone mass by androgens. The role of the androgen receptor. J Clin Invest. (1995) 95:2886–95. doi: 10.1172/JCI117995 7769130 PMC295976

[82] Chung HY Kim HJ Kim KW Choi JS Yu BP. Molecular inflammation hypothesis of aging based on the anti-aging mechanism of calorie restriction. Microsc Res Tech. (2002) 59:264–72. doi: 10.1002/jemt.10203, PMID: 12424787

[83] Iwakiri Y Sampson D Allen K. Suppression of cyclooxygenase-2 and inducible nitric oxide synthase expression by conjugated linoleic acid in murine macrophages. Prostaglandins Leukot Essent Fat Acids. (2002) 67:435–43. doi: 10.1054/plef.2002.0454, PMID: 12468265

[84] Derakhshande-Rishehri S-M Mansourian M Kelishadi R Heidari-Beni M. Association of foods enriched in conjugated linoleic acid (CLA) and CLA supplements with lipid profile in human studies: a systematic review and meta-analysis. Public Health Nutr. (2015) 18:2041–54. doi: 10.1017/S1368980014002262, PMID: 25379623 PMC10271550

[85] Gopalakrishnan S Ganeshkumar P. Systematic reviews and meta-analysis: understanding the best evidence in primary healthcare. J Family Med Prim Care. (2013) 2:9. doi: 10.4103/2249-4863.109934 24479036 PMC3894019

