# Supplementary material for: The effect of conjugated linoleic acid supplementation in comparison with omega-6 and omega-9 on lipid profile: a graded, dose–response systematic review and meta-analysis of randomized controlled trials
Source: Front Nutr. 2024 Mar 19;11:1336889. doi: 10.3389/fnut.2024.1336889 (PMC10985181; doi:10.3389/fnut.2024.1336889)

| **Supplementary Table 1**. Pooled estimates of Conjugated Linoleic Acid (CLA) supplementation among different subgroups | | | | | |
| --- | --- | --- | --- | --- | --- |
| **Group** | **Number of trials** | **WMD (95% CI)** | **P-effect** | ***I^2^* (%)** | **P- heterogeneity** |
| **TG/Olive oil** |  |  |  |  |  |
| **Linoleic Acid dosage** |  |  |  |  |  |
| < 3.4 mg/d | 6 | 0.05 (0.009, 0.10) | 0.02 | 0.0 | 0.77 |
| ≥ 3.4 mg/d | 7 | 0.001 (-0.11, 0.12) | 0.99 | 0.0 | 0.83 |
| **Duration** |  |  |  |  |  |
| ≤12 weeks | 9 | 0.05 (0.004, 0.10) | 0.03 | 0.0 | 0.97 |
| > 12 weeks | 4 | 0.03 (-0.12, 0.19) | 0.68 | 23.0 | 0.27 |
| **Mean age** |  |  |  |  |  |
| <45 years | 6 | 0.05 (0.01, 0.10) | 0.01 | 0.0 | 0.61 |
| ≥45 years | 7 | -0.02 (-0.15, 0.11 ) | 0.77 | 0.0 | 0.96 |
| **BMI** |  |  |  |  |  |
| BMI< 30 | 8 | 0.05 (0.005, 0.10) | 0.03 | 0.0 | 0.57 |
| BMI ≥ 30 | 5 | 0.01 (-0.17, 0.20) | 0.87 | 0.0 | 0.99 |
| **TG/W6** |  |  |  |  |  |
| **Linoleic Acid dosage** |  |  |  |  |  |
| < 3 mg/d | 6 | -0.02 (-0.09, 0.04) | 0.55 | 19.2 | 0.28 |
| ≥ 3 mg/d | 14 | -0.03 (-0.09, 0.02) | 0.26 | 29.1 | 0.14 |
| **Duration** |  |  |  |  |  |
| < 12 weeks | 7 | -0.05 (-0.11, 0.01) | 0.12 | 35.3 | 0.15 |
| ≥ 12 weeks | 13 | 0.002 (-0.07, 0.07) | 0.96 | 23.7 | 0.20 |
| **Mean age** |  |  |  |  |  |
| <35 years | 11 | -0.04 (-0.11, 0.03) | 0.25 | 46.4 | 0.04 |
| ≥35 years | 9 | -0.05 (-0.07, -0.03) | ˂0.001 | 0.0 | 0.60 |
| **BMI** |  |  |  |  |  |
| BMI< 25 | 7 | -0.008 (-0.11, 0.09) | 0.88 | 56.1 | 0.03 |
| BMI ≥ 25 | 12 | -0.05 (-0.07, -0.04) | ˂0.001 | 0.0 | 0.62 |
| **SEX** |  |  |  |  |  |
| Male | 6 | -0.06 (-0.12, -0.008) | 0.02 | 0.0 | 0.78 |
| Female | 4 | -0.007 (-0.11, 0.09) | 0.89 | 77.9 | 0.004 |
| Both | 10 | -0.03 (-0.11, 0.04) | 0.37 | 0.0 | 0.47 |
| **TG/placebo** |  |  |  |  |  |
| **Linoleic Acid dosage** |  |  |  |  |  |
| ≤ 3 mg/d | 5 | 0.04 (-0.09, 0.19) | 0.51 | 48.6 | 0.10 |
| > 3 mg/d | 4 | -0.02 (-0.23, 0.17) | 0.79 | 26.6 | 0.25 |
| **Duration** |  |  |  |  |  |
| < 8 weeks | 4 | 0.12 (-0.21, 0.46) | 0.47 | 58.0 | 0.06 |
| ≥ 8 weeks | 5 | -0.01 (-0.06, 0.03) | 0.48 | 0.0 | 0.52 |
| **Mean age** |  |  |  |  |  |
| <35 years | 4 | 0.03 (-0.31, 0.37) | 0.85 | 64.7 | 0.03 |
| ≥35years | 5 | -0.01 (-0.05, 0.03) | 0.62 | 0.0 | 0.50 |
| **BMI** |  |  |  |  |  |
| BMI˂25 | 4 | -0.03 (-0.20, 0.13) | 0.70 | 8.1 | 0.35 |
| BMI ≥25 | 5 | 0.07 (-0.09, 0.23) | 0.39 | 53.2 | 0.07 |
| **Tcho/olive oil** |  |  |  |  |  |
| **Linoleic Acid dosage** |  |  |  |  |  |
| < 3.4 mg/d | 5 | 0.11 (-0.08, 0.31) | 0.27 | 0.0 | 0.90 |
| ≥ 3.4 mg/d | 6 | -0.01 (-0.07, 0.04) | 0.67 | 0.0 | 0.97 |
| **Duration** |  |  |  |  |  |
| ˂12 weeks | 3 | 0.05 (-0.23, 0.35) | 0.69 | 0.0 | 0.91 |
| ≥12 weeks | 8 | -0.005 (-0.06, 0.05) | 0.85 | 0.0 | 0.89 |
| **Mean age** |  |  |  |  |  |
| <45 years | 4 | 0.06 (-0.19, 0.31) | 0.62 | 0.0 | 0.97 |
| ≥45 years | 7 | -0.006 (-0.06, 0.05) | 0.82 | 0.0 | 0.83 |
| **BMI** |  |  |  |  |  |
| BMI< 30 | 6 | 0.06 (-0.12, 0.25) | 0.50 | 0.0 | 0.99 |
| BMI ≥ 30 | 5 | -0.009 (-0.06, 0.04) | 0.75 | 0.0 | 0.66 |
| **TChol/W6** |  |  |  |  |  |
| **Linoleic Acid dosage** |  |  |  |  |  |
| ≤ 3 mg/d | 9 | 0.03 (-0.06, 0.12) | 0.51 | 10.5 | 0.34 |
| > 3 mg/d | 11 | 0.04 (-0.13, 0.21) | 0.64 | 73.1 | ˂0.001 |
| **Duration** |  |  |  |  |  |
| < 12 weeks | 7 | 0.02 (-0.08, 0.13) | 0.63 | 17.5 | 0.29 |
| ≥ 12 weeks | 13 | 0.03 (-0.13, 0.19) | 0.71 | 70.5 | ˂0.001 |
| **Mean age** |  |  |  |  |  |
| <35 years | 12 | 0.05 (-0.01, 0.12) | 0.09 | 0.0 | 0.63 |
| ≥35 years | 8 | -0.01 (-0.22, 0.20) | 0.92 | 72.6 | 0.001 |
| **BMI** |  |  |  |  |  |
| BMI< 25 | 8 | 0.04 (-0.09, 0.18) | 0.51 | 0.0 | 0.47 |
| BMI ≥ 25 | 11 | -0.01 (-0.17, 0.14) | 0.85 | 69.3 | ˂0.001 |
| **SEX** |  |  |  |  |  |
| Male | 6 | 0.07 (0.001, 0.15) | 0.04 | 0.0 | 0.92 |
| Female | 4 | -0.01 (-0.33, 0.29) | 0.91 | 85.4 | ˂0.001 |
| Both | 10 | -0.01 (-0.13, 0.10) | 0.81 | 4.9 | 0.39 |
| **Tcho/placebo** |  |  |  |  |  |
| **Linoleic Acid dosage** |  |  |  |  |  |
| ≤ 3 mg/d | 3 | -0.05 (-0.29, 0.18) | 0.66 | 0.0 | 0.99 |
| > 3 mg/d | 4 | -0.08 (-0.15, -0.01) | 0.01 | 91.2 | ˂0.001 |
| **Duration** |  |  |  |  |  |
| ˂8 weeks | 4 | -0.02 (-0.05, 0.002) | 0.06 | 46.5 | 0.13 |
| ≥8 weeks | 3 | -0.22 (-0.29, -0.14) | ˂0.001 | 0.0 | 0.44 |
| **Mean age** |  |  |  |  |  |
| ≤ 25 years | 4 | -0.02 (-0.06, 0.009) | 0.13 | 64.8 | 0.03 |
| > 25 years | 3 | -0.21 (-0.28, -0.14) | ˂0.001 | 0.0 | 0.72 |
| **BMI** |  |  |  |  |  |
| BMI< 25 | 4 | -0.02 (-0.06, 0.009) | 0.13 | 64.9 | 0.03 |
| BMI ≥ 25 | 3 | -0.20 (-0.28, -0.13) | ˂0.001 | 0.0 | 0.49 |
| **LDL/olive oil** |  |  |  |  |  |
| **Linoleic Acid dosage** |  |  |  |  |  |
| < 3.4 mg/d | 5 | 0.02 (-0.07, 0.12) | 0.67 | 0.0 | 0.92 |
| ≥ 3.4 mg/d | 6 | 0.11 (-0.04, 0.26) | 0.15 | 0.0 | 0.92 |
| **Duration** |  |  |  |  |  |
| ˂12 weeks | 3 | 0.01 (-0.09, 0.12) | 0.79 | 0.0 | 0.74 |
| ≥12 weeks | 8 | 0.10 (-0.03, 0.23) | 0.13 | 0.0 | 0.97 |
| **Mean age** |  |  |  |  |  |
| <45 years | 4 | 0.01 (-0.08, 0.12) | 0.74 | 0.0 | 0.83 |
| ≥45 years | 7 | 0.09 (-0.03, 0.23) | 0.15 | 0.0 | 0.95 |
| **BMI** |  |  |  |  |  |
| BMI< 30 | 9 | 0.03 (-0.05, 0.12) | 0.44 | 0.0 | 0.97 |
| BMI ≥ 30 | 2 | 0.17 (-0.08, 0.43) | 0.18 | 0.0 | 0.80 |
| **LDL/W6** |  |  |  |  |  |
| **Linoleic Acid dosage** |  |  |  |  |  |
| < 3.4 g/d | 6 | -0.16 (-0.29, -0.03) | 0.01 | 63.1 | 0.01 |
| ≥ 3.4 g/d | 7 | 0.08 (-0.05, 0.22) | 0.24 | 0.0 | 0.98 |
| **Duration** |  |  |  |  |  |
| < 12weeks | 2 | -0.13 (-0.74, 0.47) | 0.65 | 67.4 | 0.08 |
| ≥ 12weeks | 11 | -0.04 (-0.16, 0.07) | 0.43 | 63.7 | 0.002 |
| **Mean age** |  |  |  |  |  |
| <40 years | 6 | 0.03 (-0.08, 0.14) | 0.56 | 0.0 | 0.92 |
| ≥40 years | 7 | -0.15 (-0.27, -0.02) | 0.01 | 46.3 | 0.08 |
| **BMI** |  |  |  |  |  |
| BMI< 30 | 9 | -0.08 (-0.22, 0.05) | 0.22 | 65.0 | 0.004 |
| BMI ≥ 30 | 4 | -0.01 (-0.16, 0.14) | 0.87 | 0.0 | 0.53 |
| **SEX** |  |  |  |  |  |
| Male | 4 | -0.03 (-0.19, 0.13) | 0.71 | 0.0 | 0.70 |
| Female | 3 | -0.12 (-0.39, 0.14) | 0.35 | 49.0 | 0.14 |
| Both | 6 | -0.04 (-0.17, 0.09) | 0.52 | 30.1 | 0.20 |
| **LDL/placebo** |  |  |  |  |  |
| **Linoleic Acid dosage** |  |  |  |  |  |
| ≤ 3 mg/d | 5 | 0.04 (-0.10, 0.20) | 0.53 | 55.5 | 0.06 |
| > 3 mg/d | 4 | -0.27 (-0.51, -0.03) | 0.023 | 56.0 | 0.07 |
| **Duration** |  |  |  |  |  |
| ˂8 weeks | 4 | 0.05 (-0.26, 0.36) | 0.7 | 51.5 | 0.10 |
| ≥8 weeks | 5 | -0.13 (-0.37, 0.10) | 0.28 | 92.3 | ˂0.001 |
| **Mean age** |  |  |  |  |  |
| <35years | 4 | 0.01 (-0.30, 0.33) | 0.93 | 64.4 | 0.03 |
| ≥35 years | 5 | -0.12 (-0.37, 0.12) | 0.33 | 92.3 | ˂0.001 |
| **BMI** |  |  |  |  |  |
| BMI˂25 | 4 | -0.04 (-0.23, 0.13) | 0.61 | 0.0 | 0.50 |
| BMI ≥ 25 | 5 | -0.06 (-0.34, 0.21) | 0.63 | 94.5 | ˂0.001 |
| **HDL/Olive oil** |  |  |  |  |  |
| **Linoleic Acid dosage** |  |  |  |  |  |
| ˂3.4 mg/d | 5 | 0.032 (-0.01, 0.07) | 0.15 | 0.0 | 0.64 |
| ≥ 3.4 mg/d | 6 | -0.07 (-0.11, -0.03) | ˂0.001 | 0.0 | 0.95 |
| **Duration** |  |  |  |  |  |
| ˂12 weeks | 3 | -0.002 (-0.09, 0.09) | 0.96 | 66.2 | 0.05 |
| ≥12 weeks | 8 | -0.052 (-0.08, -0.01) | 0.005 | 0.0 | 0.76 |
| **Mean age** |  |  |  |  |  |
| <45 years | 4 | 0.01 (-0.06, 0.09) | 0.69 | 21.2 | 0.28 |
| ≥45 years | 7 | -0.05 (-0.08, -0.01) | 0.003 | 0.0 | 0.67 |
| **BMI** |  |  |  |  |  |
| BMI˂30 | 6 | 0.02 (-0.01, 0.06) | 0.29 | 0.0 | 0.56 |
| BMI ≥ 30 | 5 | 0.06 (-0.10, -0.02) | 0.001 | 0.0 | 0.71 |
| **SEX** |  |  |  |  |  |
| Male | 3 | -0.07 (-0.11, -0.02) | 0.005 | 0.0 | 0.37 |
| Female | 2 | -0.03 (-0.17, 0.10) | 0.65 | 0.0 | 0.76 |
| Both | 6 | -0.01 (-0.06, 0.04) | 0.69 | 40.0 | 0.13 |
| **HDL/W6** |  |  |  |  |  |
| **Linoleic Acid dosage** |  |  |  |  |  |
| < 3 g/d | 5 | -0.02 (-0.07, 0.02) | 0.34 | 0.0 | 0.62 |
| ≥ 3 g/d | 12 | -0.01 ( -0.02, 0.008) | 0.26 | 0.0 | 0.60 |
| **Duration** |  |  |  |  |  |
| < 12weeks | 4 | -0.006 (-0.09, 0.08) | 0.90 | 41.4 | 0.16 |
| ≥ 12weeks | 13 | -0.01 (-0.02, 0.005) | 0.15 | 0.0 | 0.86 |
| **Mean age** |  |  |  |  |  |
| <35 years | 8 | -0.01 (-0.04, 0.02) | 0.52 | 0.0 | 0.74 |
| ≥35 years | 9 | -0.01 (-0.03, 0.007) | 0.23 | 0.0 | 0.44 |
| **BMI** |  |  |  |  |  |
| BMI< 25 | 6 | -0.01 (-0.07, 0.05) | 0.68 | 20.4 | 0.28 |
| BMI ≥ 25 | 11 | -0.01 (-0.02, 0.006) | 0.18 | 0.0 | 0.82 |
| **SEX** |  |  |  |  |  |
| Male | 5 | -0.02 (-0.08, 0.04) | 0.52 | 0.0 | 0.78 |
| Female | 4 | -0.02 (-0.03, 0.000) | 0.05 | 0.0 | 0.85 |
| Both | 8 | 0.01 (-0.01, 0.04) | 0.34 | 0.0 | 0.48 |
| **HDL/placebo** |  |  |  |  |  |
| **Linoleic Acid dosage** |  |  |  |  |  |
| ≤ 3 mg/d | 4 | 0.03 (-0.04, 0.10) | 0.41 | 19.3 | 0.29 |
| > 3 mg/d | 4 | -0.04 (-0.26, 0.18) | 0.71 | 75.4 | 0.007 |
| **Duration** |  |  |  |  |  |
| ˂8 weeks | 4 | -0.06 (-0.35, 0.21) | 0.64 | 68.1 | 0.02 |
| ≥8 weeks | 4 | 0.04 (-0.05, 0.13) | 0.39 | 71.9 | 0.01 |
| **Mean age** |  |  |  |  |  |
| <30 years | 5 | -0.02 (-0.15, 0.10) | 0.69 | 42.3 | 0.14 |
| ≥30 years | 3 | 0.08 (-0.02, 0.19) | 0.13 | 72.9 | 0.02 |
| **BMI** |  |  |  |  |  |
| BMI˂ 25 | 4 | 0.08 (-0.25, 0.08) | 0.33 | 49.8 | 0.11 |
| BMI ≥ 25 | 4 | 0.09 (0.03, 0.16) | 0.002 | 30.9 | 0.22 |
| **SEX** |  |  |  |  |  |
| Male | 2 | -0.008 (-0.12, 0.10) | 0.89 | 0.0 | 0.80 |
| Female | 2 | -0.75 (-1.33, -0.17) | 0.01 | 0.0 | 0.78 |
| Both | 4 | 0.07 (-0.01, 0.15) | 0.10 | 67.3 | 0.02 |
| Abbreviations. | | | | | |

**Supplemental Table 2**: Results of risk of bias assessment for randomized clinical trials included in the current meta-analysis on the effects of Conjugated Linoleic Acid (CLA) supplementation on lipid profile.

| **Study** | Random sequence generation | Allocation concealment | Selective reporting | Blinding (participants and personnel) | Blinding (outcome assessment) | Incomplete outcome data | other source of bias | Overall Quality |
| --- | --- | --- | --- | --- | --- | --- | --- | --- |
| Blankson (2000) | L | L | L | L | U | L | L | Fair |
| Breven (2000) | L | U | L | L | U | L | L | Fair |
| Benito (2001) | L | L | L | H | H | L | L | Poor |
| Mougios (2001) | H | U | L | L | U | L | L | Fair |
| Rise´rus (2001) | L | U | L | L | L | L | L | Fair |
| Rise´rus (2002) | L | U | L | L | L | L | L | Fair |
| Noone (2002) | L | U | L | L | U | L | L | Fair |
| Kamphuis (2003) | L | U | L | L | U | L | H | Fair |
| Petridou (2003) | L | U | L | L | U | L | H | Fair |
| Risérus (2004) | L | U | L | L | U | L | L | Fair |
| Moloney (2004) | L | U | L | L | U | L | L | Fair |
| Whigham (2004) | L | U | L | L | U | L | L | Fair |
| Gaullier 2005 | L | L | L | L | L | L | L | Good |
| Song (2005) | L | U | H | L | U | L | L | Fair |
| Colakoglu (2006) | L | U | L | L | H | L | L | Fair |
| Taylor (2006) | L | U | U | L | U | L | L | Fair |
| Watras (2006) | L | L | L | L | U | L | L | Fair |
| Iwata (2007) | L | U | L | L | U | L | L | Fair |
| Lambert (2007) | L | U | L | L | U | L | L | Fair |
| Steck (2007) | L | U | L | L | U | L | L | Fair |
| Aryaeian (2008) | L | U | L | L | U | L | L | Fair |
| Kim (2008) | L | U | L | L | U | L | L | Fair |
| Park (2008) | L | U | L | L | U | L | L | Fair |
| Zhao (2009) | L | U | L | L | U | L | L | Fair |
| Tavakoli-Darestabi (2010) | L | U | L | L | U | L | L | Fair |
| Joseph (2011) | L | U | L | L | U | L | L | Fair |
| pfeuffer (2011) | L | U | L | L | U | L | L | Fair |
| Bulut (2013) | L | U | L | L | U | L | H | Fair |
| Eftekhari (2014) | L | U | L | H | H | L | L | Poor |
| Jenkins (2014) | L | U | L | L | U | L | L | Fair |
| Baghi (2016) | L | U | L | L | U | L | H | Fair |
| Ribeiro (2016) | L | U | L | L | U | H | H | Poor |
| Fouladi (2018) | L | U | L | H | H | L | L | Poor |
| chang (2020) | L | U | L | L | U | L | L | Fair |
| mahdavi (2021) | L | U | L | H | H | L | L | Poor |

U; unclear risk of bias, L; low risk of bias, H; high risk of bias

**Supplementary Table 3**: GRADE evidence table for the effects of conjugated linoleic acid on blood lipids.

| **Certainty assessment** | | | | | | | **№ of patients** | | **Mean difference (95%CI)** | **Certainty** | **Importance** |
| --- | --- | --- | --- | --- | --- | --- | --- | --- | --- | --- | --- |
| **№ of studies** | **Study design** | **Risk of bias** | **Inconsistency** | **Indirectness** | **Imprecision** | **Other considerations** | **[intervention]** | **[comparison]** |  |  |  |
| **CLA supplement vs olive oil on TG** | | | | | | | | | | | |
| 11 | randomised trials | serious^a^ | not serious | not serious | serious^b^ | none | 233 | 181 | MD **0.05 mmol/L higher** (0 to 0.1 higher) | ⨁⨁◯◯ Low | IMPORTANT |
| **CLA supplement vs omega-6 on TG** | | | | | | | | | | | |
| 15 | randomised trials | serious^a^ | not serious | not serious | serious^b^ | none | 330 | 278 | MD **0.03 mmol/L lower** (0.08 lower to 0.01 higher) | ⨁⨁◯◯ Low | IMPORTANT |
| **CLA supplement vs placebo on TG** | | | | | | | | | | | |
| 8 | randomised trials | serious^a^ | not serious | not serious | serious^b^ | none | 231 | 179 | MD **0.02 mmol/L higher** (0.09 lower to 0.12 higher) | ⨁⨁◯◯ Low | IMPORTANT |
| **CLA supplement vs olive oil on TC** | | | | | | | | | | | |
| 10 | randomised trials | serious^a^ | not serious | not serious | serious^c^ | none | 216 | 186 | MD **0 mmol/L**  (0.06 lower to 0.05 higher) | ⨁⨁◯◯ Low | IMPORTANT |
| **CLA supplement vs omega-6 on TC** | | | | | | | | | | | |
| 15 | randomised trials | serious^a^ | not serious | not serious | serious^c^ | none | 330 | 278 | MD **0.02 mmol/L lower** (0.15 lower to 0.12 higher) | ⨁⨁◯◯ Low | IMPORTANT |
| **CLA supplement vs placebo on TC** | | | | | | | | | | | |
| 6 | randomised trials | serious^a^ | serious^d^ | not serious | serious^c^ | none | 139 | 114 | MD **0.08 mmol/L lower** (0.14 lower to 0.02 lower) | ⨁◯◯◯ Very low | IMPORTANT |
| **CLA supplement vs olive oil on LDL** | | | | | | | | | | | |
| 10 | randomised trials | serious^a^ | not serious | not serious | serious^e^ | none | 215 | 187 | MD **0.05 mmol/L higher** (0.03 lower to 0.13 higher) | ⨁⨁◯◯ Low | IMPORTANT |
| **CLA supplement vs omega-6 on LDL** | | | | | | | | | | | |
| 11 | randomised trials | serious^a^ | serious^f^ | not serious | serious^e^ | none | 225 | 193 | MD **0.06 mmol/L lower** (0.18 lower to 0.05 higher) | ⨁◯◯◯ Very low | IMPORTANT |
| **CLA supplement vs placebo on LDL** | | | | | | | | | | | |
| 8 | randomised trials | serious^a^ | serious^g^ | not serious | serious^e^ | none | 187 | 156 | MD **0.07 mmol/L lower** (0.29 lower to 0.14 higher) | ⨁◯◯◯ Very low | IMPORTANT |
| **CLA supplement vs olive oil on HDL** | | | | | | | | | | | |
| 10 | randomised trials | serious^a^ | not serious | not serious | serious^h^ | none | 215 | 187 | MD **0.03 mmol/L lower** (0.07 lower to 0.01 higher) | ⨁⨁◯◯ Low | IMPORTANT |
| **CLA supplement vs omega-6 on HDL** | | | | | | | | | | | |
| 13 | randomised trials | serious^a^ | not serious | not serious | serious^h^ | none | 287 | 259 | MD **0.01 mmol/L lower** (0.03 lower to 0 ) | ⨁⨁◯◯ Low | IMPORTANT |
| **CLA supplement vs placebo on HDL** | | | | | | | | | | | |
| 7 | randomised trials | serious^a^ | serious^i^ | not serious | serious^h^ | none | 187 | 156 | MD **0.03 mmol/L lower** (0.05 lower to 0.02 higher) | ⨁◯◯◯ Very low | IMPORTANT |

**CI:** confidence interval; **MD:** mean difference

#### Explanations

a. Serious risk of bias due to allocation concealment and blinding. Downgraded.

b. Serious imprecision since the point estimate was smaller than the MCID for TG (0.09 mmol/L). Downgraded.

c. Serious imprecision since the point estimate was smaller than the MCID for TC (0.26 mmol/L). Downgraded.

d. Serious inconsistency since I^2^=86%. Downgraded.

e. Serious imprecision since the point estimate was smaller than the MCID for LDL (0.10 mmol/L). Downgraded.

f. Serious inconsistency since I^2^=61%. Downgraded.

g. Serious inconsistency since I^2^=90%. Downgraded.

h. Serious imprecision since the point estimate was smaller than the MCID for HDL (0.10 mmol/L). Downgraded.

i. Serious inconsistency since I^2^=66%. Downgraded.

**Supplementary Figure1:** Forest plot for the effect of CLA supplementation versus Omega-6 on serum levels of TG, expressed as weighted mean differences between intervention and control groups.


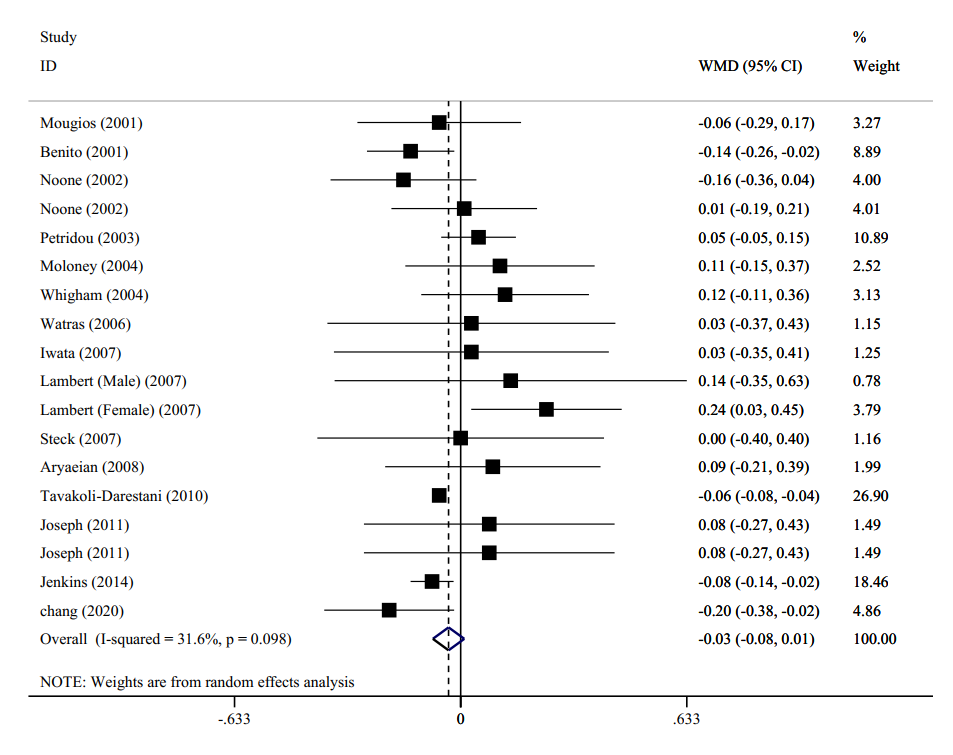


**Supplementary Figure2:** Forest plot for the effect of CLA supplementation versus placebo on serum levels of TG, expressed as weighted mean differences between intervention and control groups.


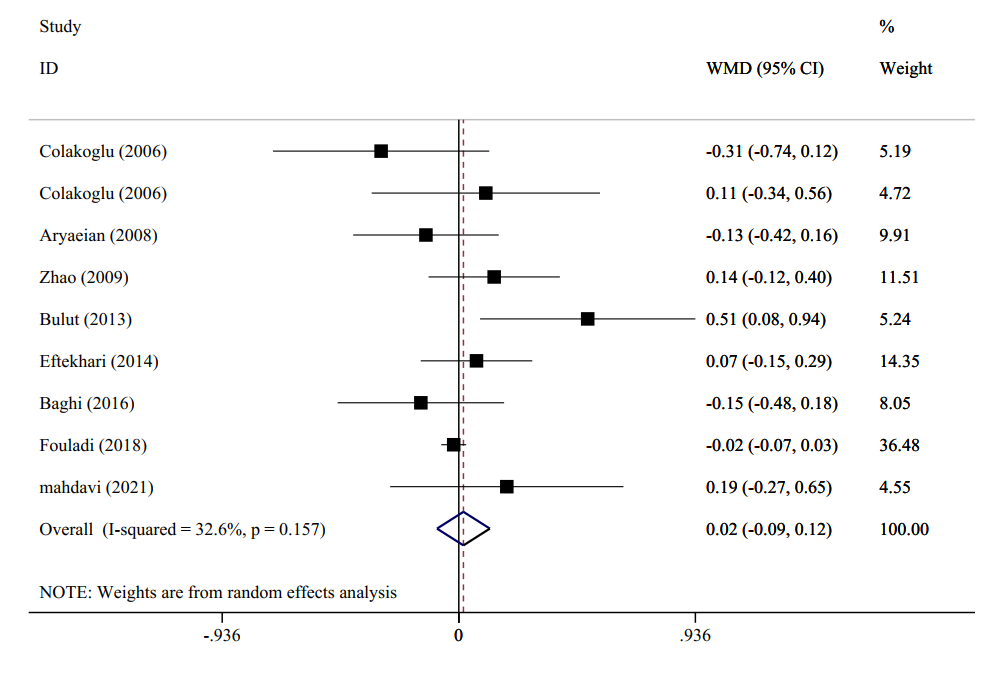


**Supplementary Figure 3:** Forest plot for the effect of CLA supplementation versus olive oil on serum levels of total cholesterol, expressed as weighted mean differences between intervention and control groups.


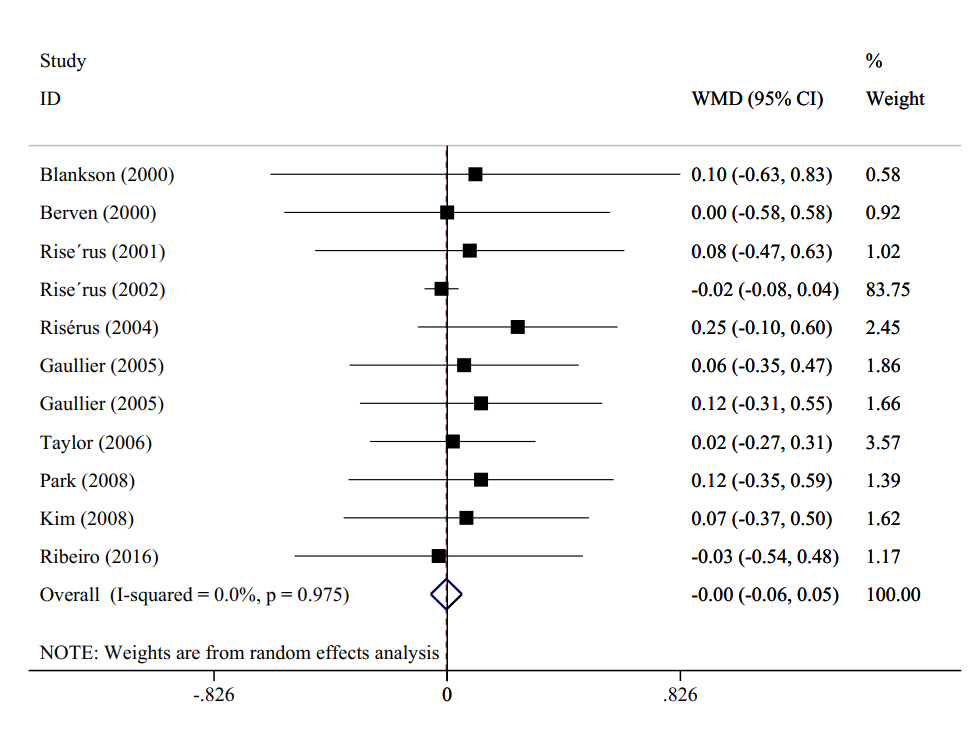


**Supplementary Figure 4:** Forest plot for the effect of CLA supplementation versus W6 on serum levels of total cholesterol, expressed as weighted mean differences between intervention and control groups.


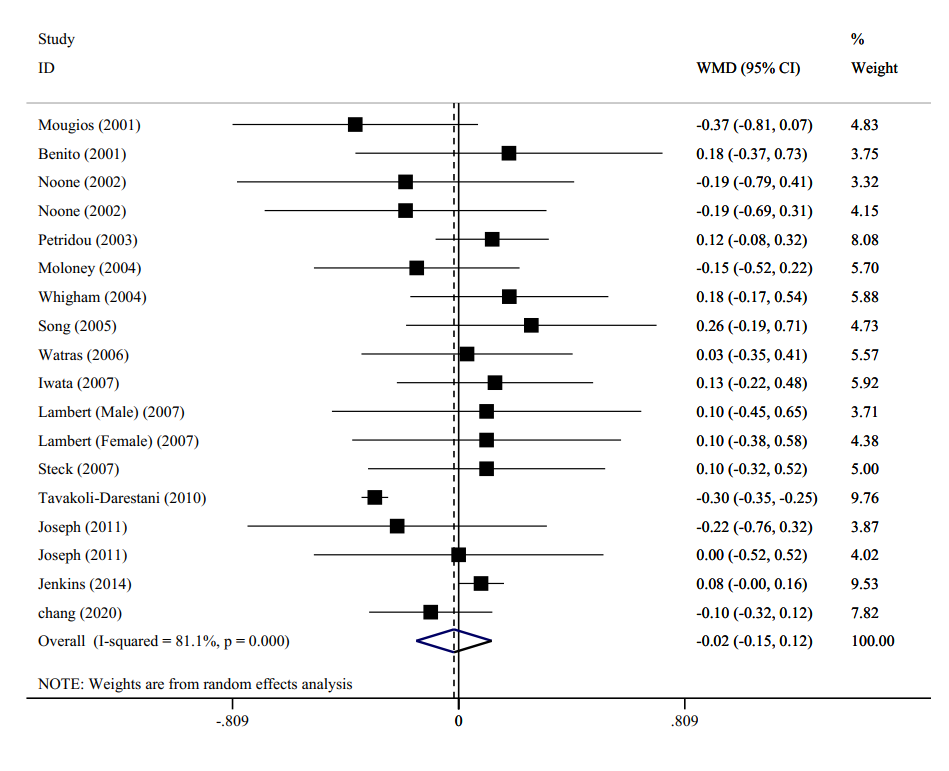


**Supplementary Figure 5:** Forest plot for the effect of CLA supplementation versus olive oil on serum levels of LDL, expressed as weighted mean differences between intervention and control groups.


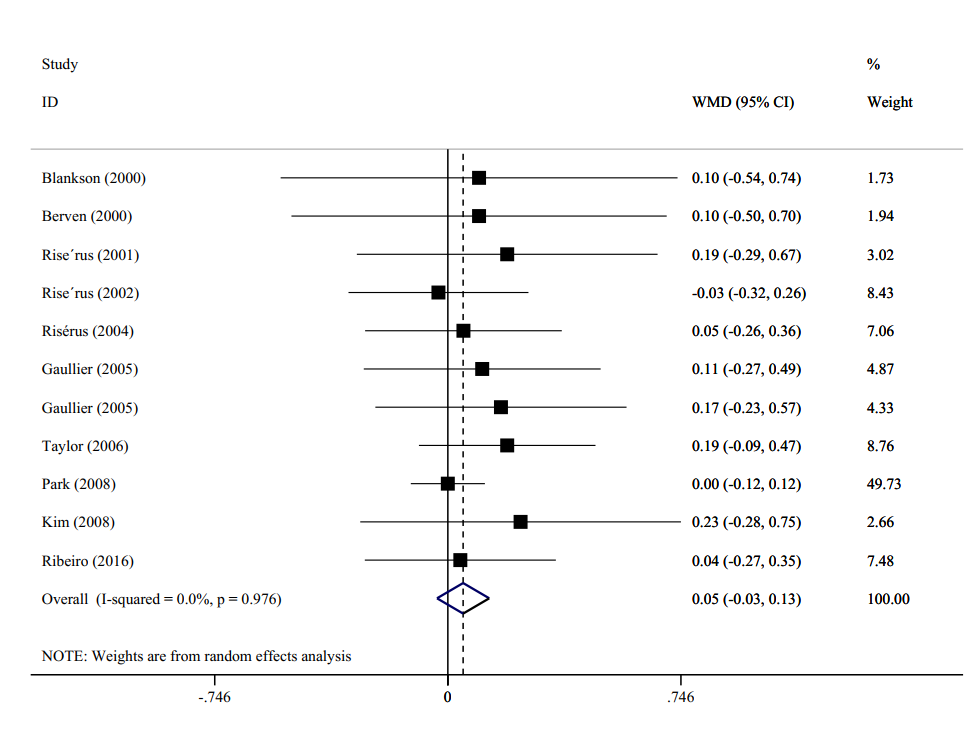


**Supplementary Figure 6:** Forest plot for the effect of CLA supplementation versus W6 on serum levels of LDL, expressed as weighted mean differences between intervention and control groups.


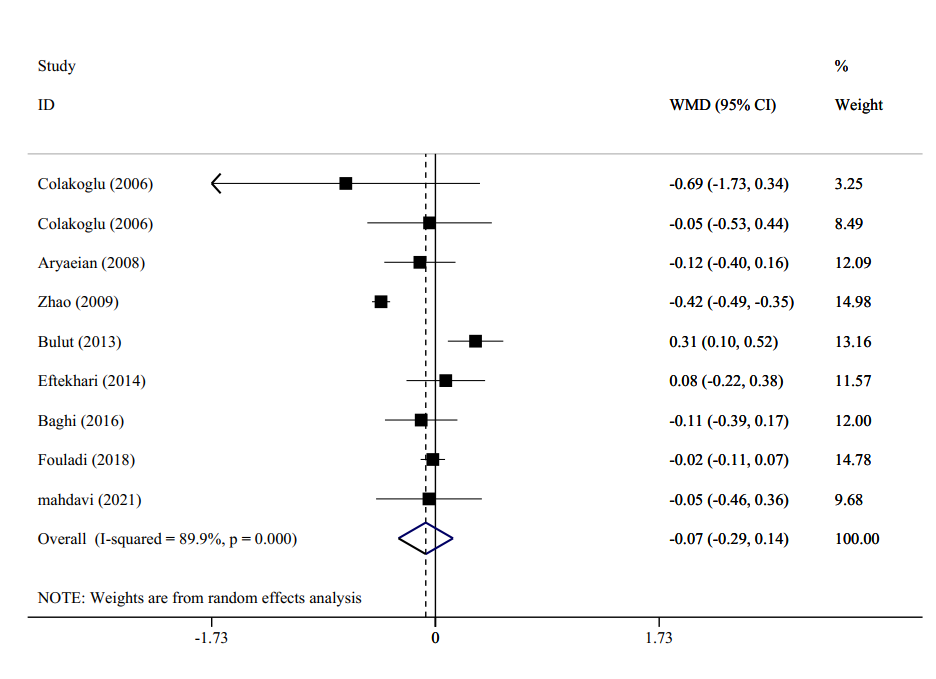


**Supplementary Figure 7:** Forest plot for the effect of CLA supplementation versus placebo on serum levels of LDL, expressed as weighted mean differences between intervention and control groups.


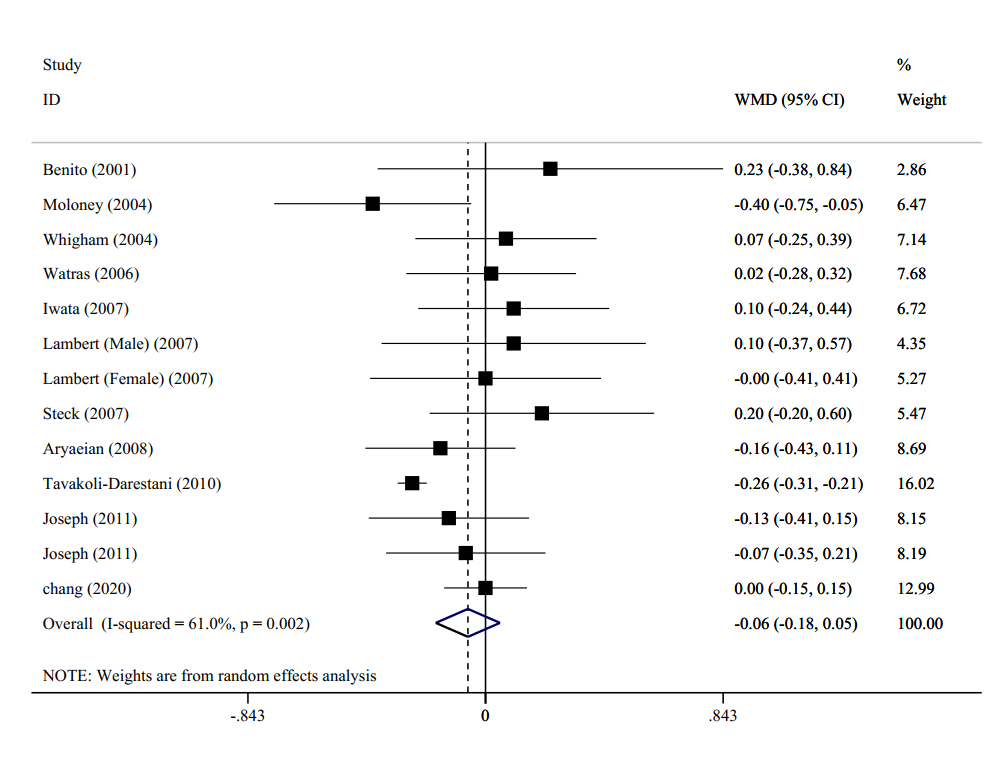


**Supplementary Figure 8:** Forest plot for the effect of CLA supplementation versus olive oil on serum levels of HDL, expressed as weighted mean differences between intervention and control groups.


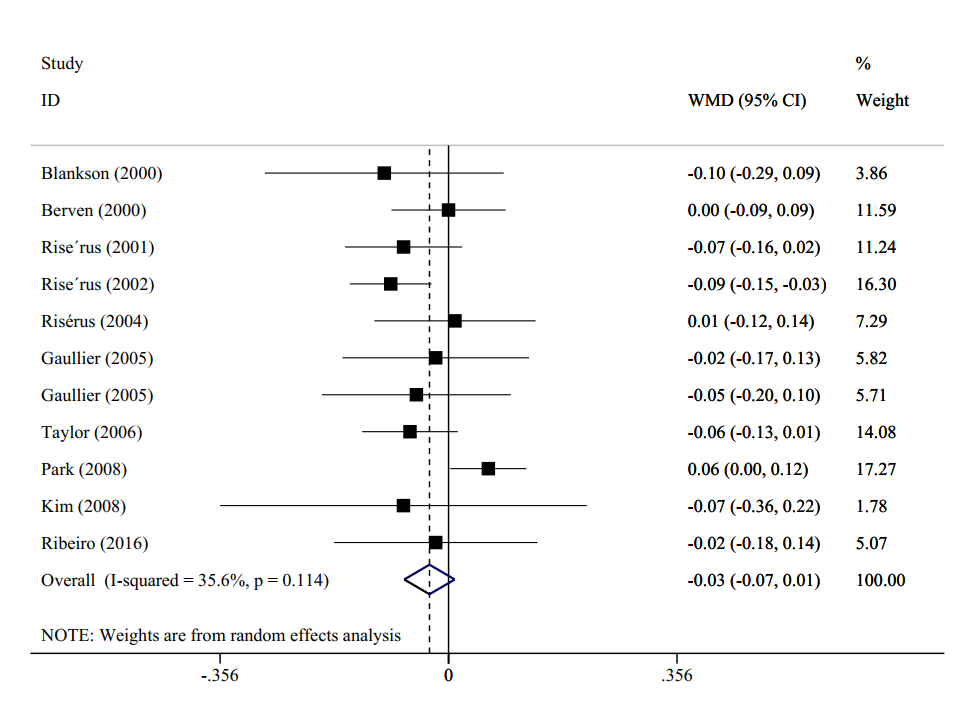


**Supplementary Figure 9:** Forest plot for the effect of CLA supplementation versus W6 on serum levels of HDL, expressed as weighted mean differences between intervention and control groups.


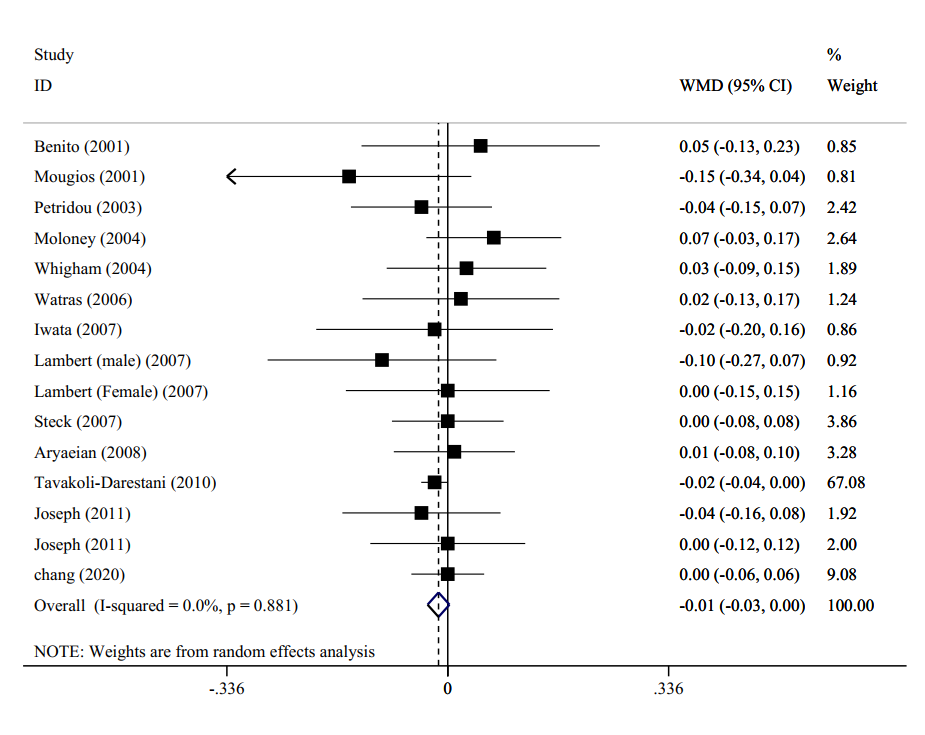


**Supplementary Figure 10:** Forest plot for the effect of CLA supplementation versus placebo on serum levels of HDL, expressed as weighted mean differences between intervention and control groups.


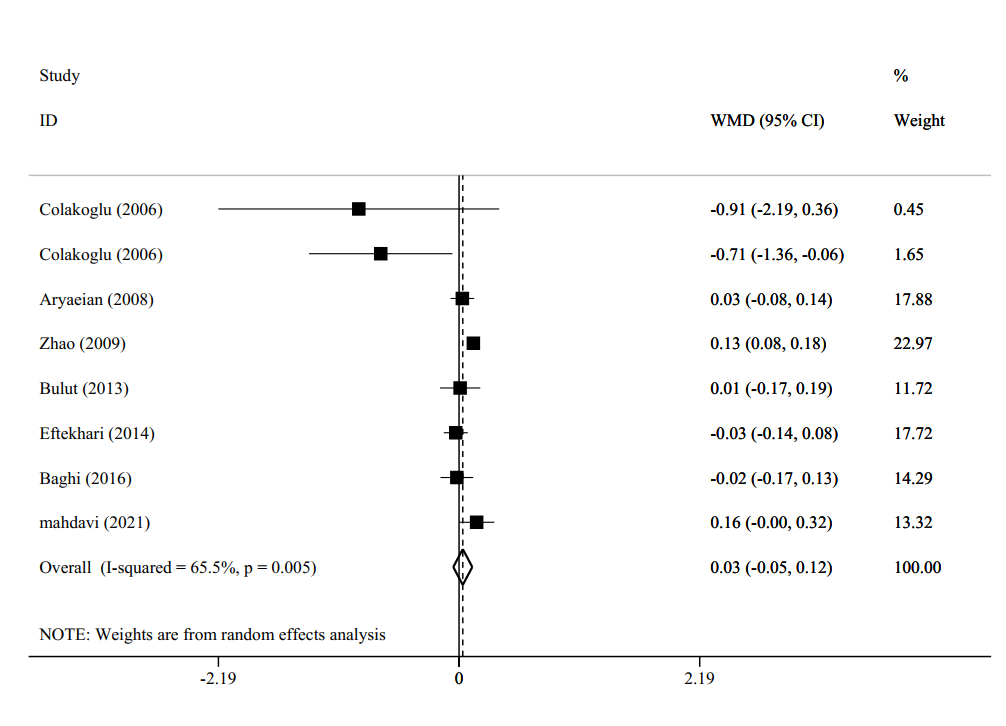

Supplement: Supplementary file 1 [file Data_Sheet_1.docx]
